# Supplementary material for: A yeast phenomic model for the influence of Warburg metabolism on genetic buffering of doxorubicin
Source: Cancer Metab. 2019 Oct 23;7:9. doi: 10.1186/s40170-019-0201-3 (PMC6806529; doi:10.1186/s40170-019-0201-3)

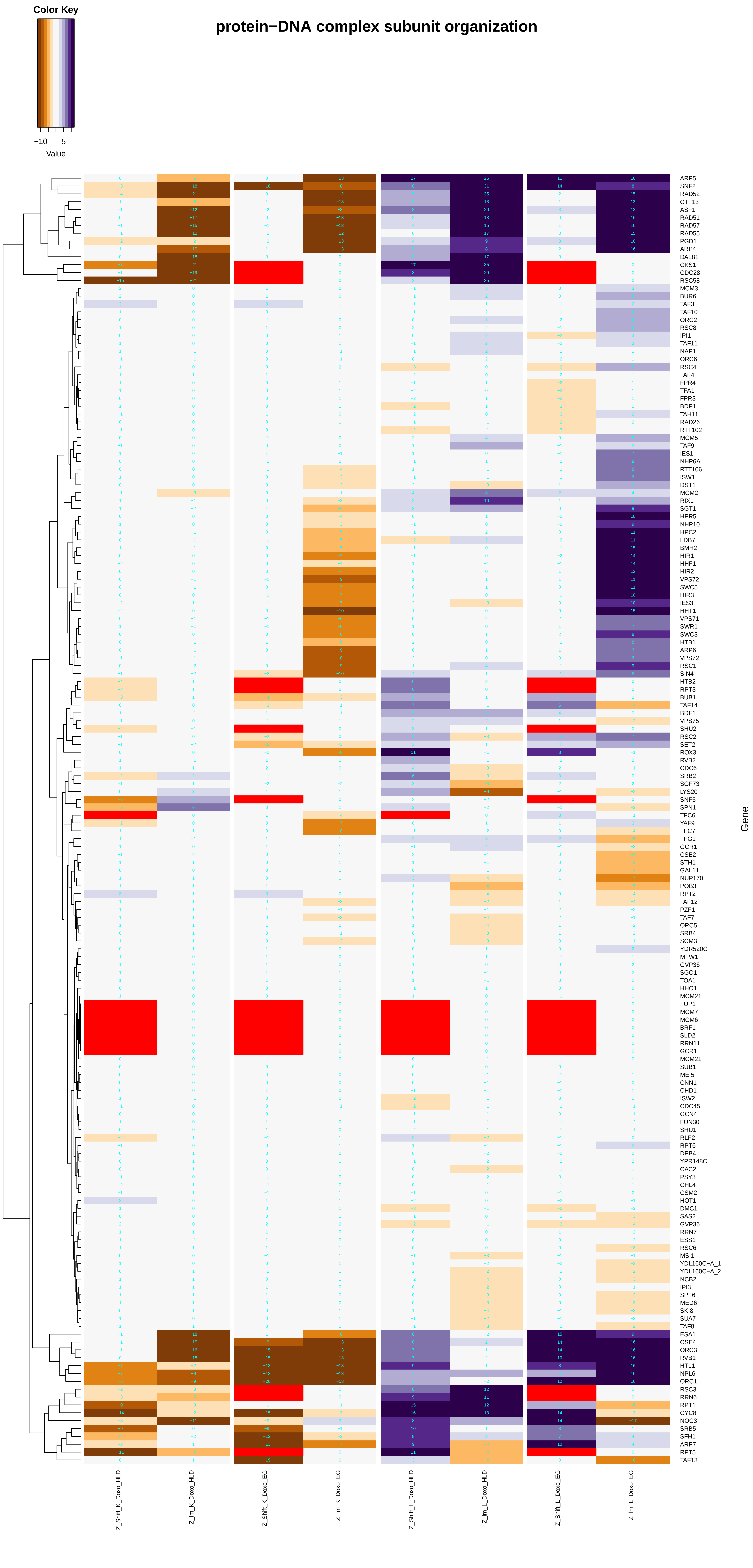
Color Key
−10
5
Value
protein−DNA complex subunit organization
ARP5
SNF2
RAD52
CTF13
ASF1
RAD51
RAD57
RAD55
PGD1
ARP4
DAL81
CKS1
CDC28
RSC58
MCM3
BUR6
TAF3
TAF10
ORC2
RSC8
IPI1
TAF11
NAP1
ORC6
RSC4
TAF4
FPR4
TFA1
FPR3
BDP1
TAH11
RAD26
RTT102
MCM5
TAF9
IES1
NHP6A
RTT106
ISW1
DST1
MCM2
RIX1
SGT1
HPR5
NHP10
HPC2
LDB7
BMH2
HIR1
HHF1
HIR2
VPS72
SWC5
HIR3
IES3
HHT1
VPS71
SWR1
SWC3
HTB1
ARP6
VPS72
RSC1
SIN4
HTB2
RPT3
BUB1
TAF14
BDF1
VPS75
SHU2
RSC2
SET2
ROX3
RVB2
CDC6
SRB2
SGF73
LYS20
SNF5
SPN1
TFC6
YAF9
TFC7
TFG1
GCR1
CSE2
STH1
GAL11
NUP170
POB3
RPT2
TAF12
PZF1
TAF7
ORC5
SRB4
SCM3
YDR520C
MTW1
GVP36
SGO1
TOA1
HHO1
MCM21
TUP1
MCM7
MCM6
BRF1
SLD2
RRN11
GCR1
MCM21
SUB1
MEI5
CNN1
CHD1
ISW2
CDC45
GCN4
FUN30
SHU1
RLF2
RPT6
DPB4
YPR148C
CAC2
PSY3
CHL4
CSM2
HOT1
DMC1
SAS2
GVP36
RRN7
ESS1
RSC6
MSI1
YDL160C−A_1
YDL160C−A_2
NCB2
IPI3
SPT6
MED6
SKI8
SUA7
TAF8
ESA1
CSE4
ORC3
RVB1
HTL1
NPL6
ORC1
RSC3
RRN6
RPT1
CYC8
NOC3
SRB5
SFH1
ARP7
RPT5
TAF13
Gene
Z_Shift_K_Doxo_HLD
Z_lm_K_Doxo_HLD
Z_Shift_K_Doxo_EG
Z_lm_K_Doxo_EG
Z_Shift_L_Doxo_HLD
Z_lm_L_Doxo_HLD
Z_Shift_L_Doxo_EG
Z_lm_L_Doxo_EG

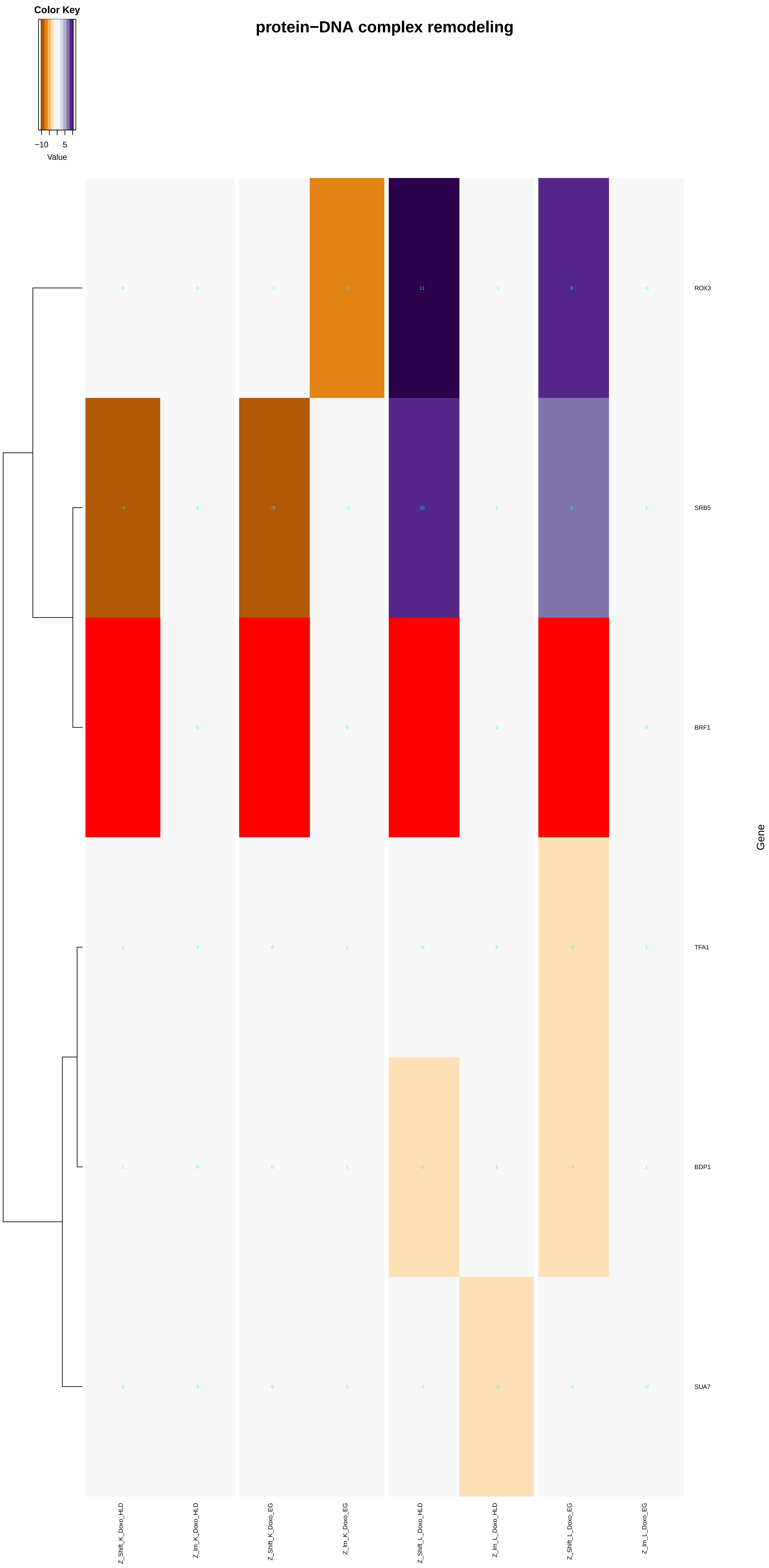
Color Key
−10 5
Value
protein−DNA complex remodeling
ROX3
SRB5
BRF1
TFA1
BDP1
SUA7
Gene
Z_Shift_K_Doxo_HLD
Z_Im_K_Doxo_HLD
Z_Shift_K_Doxo_EG
Z_Im_K_Doxo_EG
Z_Shift_L_Doxo_HLD
Z_Im_L_Doxo_HLD
Z_Shift_L_Doxo_EG
Z_Im_L_Doxo_EG

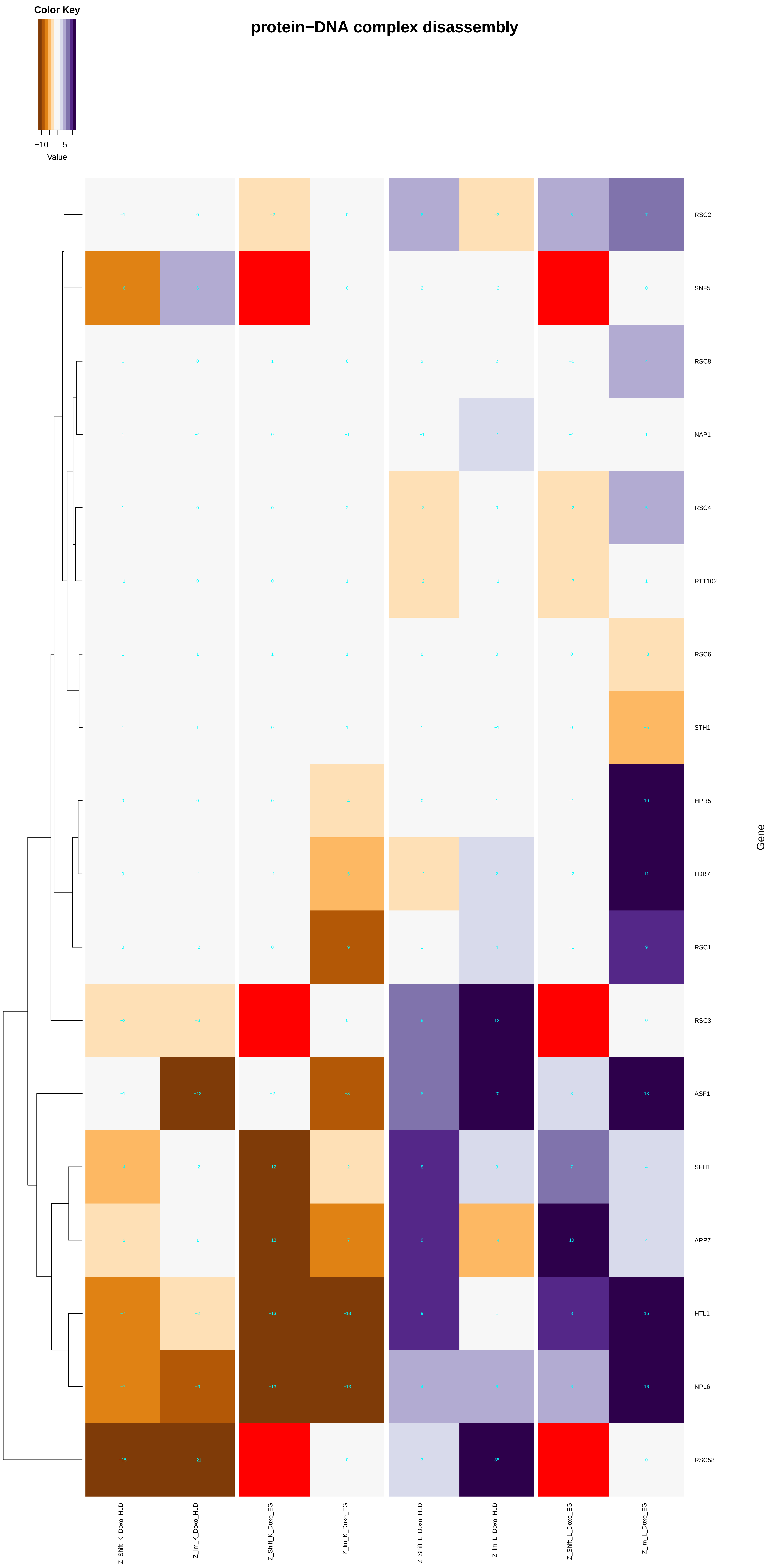
protein−DNA complex disassembly
Color Key
−10
5
Value
RSC2
SNF5
RSC8
NAP1
RSC4
RTT102
RSC6
STH1
HPR5
LDB7
RSC1
RSC3
ASF1
SFH1
ARP7
HTL1
NPL6
RSC58
Gene
Z_Shift_K_Doxo_HLD
Z_lm_K_Doxo_HLD
Z_Shift_K_Doxo_EG
Z_lm_K_Doxo_EG
Z_Shift_L_Doxo_HLD
Z_lm_L_Doxo_HLD
Z_Shift_L_Doxo_EG
Z_lm_L_Doxo_EG

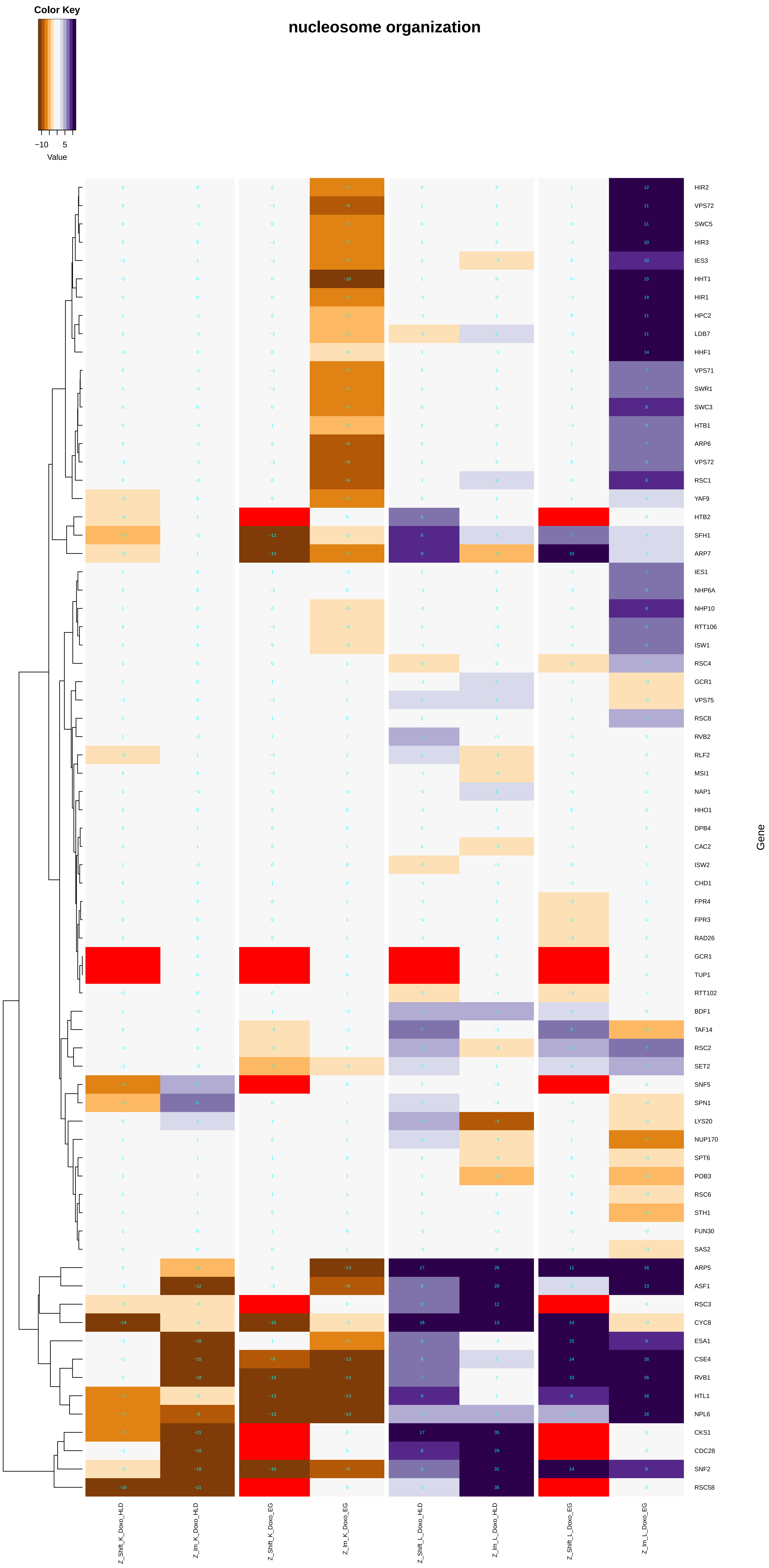
Color Key
−10 5
Value
nucleosome organization
HIR2
VPS72
SWC5
HIR3
IES3
HHT1
HIR1
HPC2
LDB7
HHF1
VPS71
SWR1
SWC3
HTB1
ARP6
VPS72
RSC1
YAF9
HTB2
SFH1
ARP7
IES1
NHP6A
NHP10
RTT106
ISW1
RSC4
GCR1
VPS75
RSC8
RVB2
RLF2
MSI1
NAP1
HHO1
DPB4
CAC2
ISW2
CHD1
FPR4
FPR3
RAD26
GCR1
TUP1
RTT102
BDF1
TAF14
RSC2
SET2
SNF5
SPN1
LYS20
NUP170
SPT6
POB3
RSC6
STH1
FUN30
SAS2
ARP5
ASF1
RSC3
CYC8
ESA1
CSE4
RVB1
HTL1
NPL6
CKS1
CDC28
SNF2
RSC58
Gene
Z_Shift_K_Doxo_HLD
Z_Im_K_Doxo_HLD
Z_Shift_K_Doxo_EG
Z_Im_K_Doxo_EG
Z_Shift_L_Doxo_HLD
Z_Im_L_Doxo_HLD
Z_Shift_L_Doxo_EG
Z_Im_L_Doxo_EG

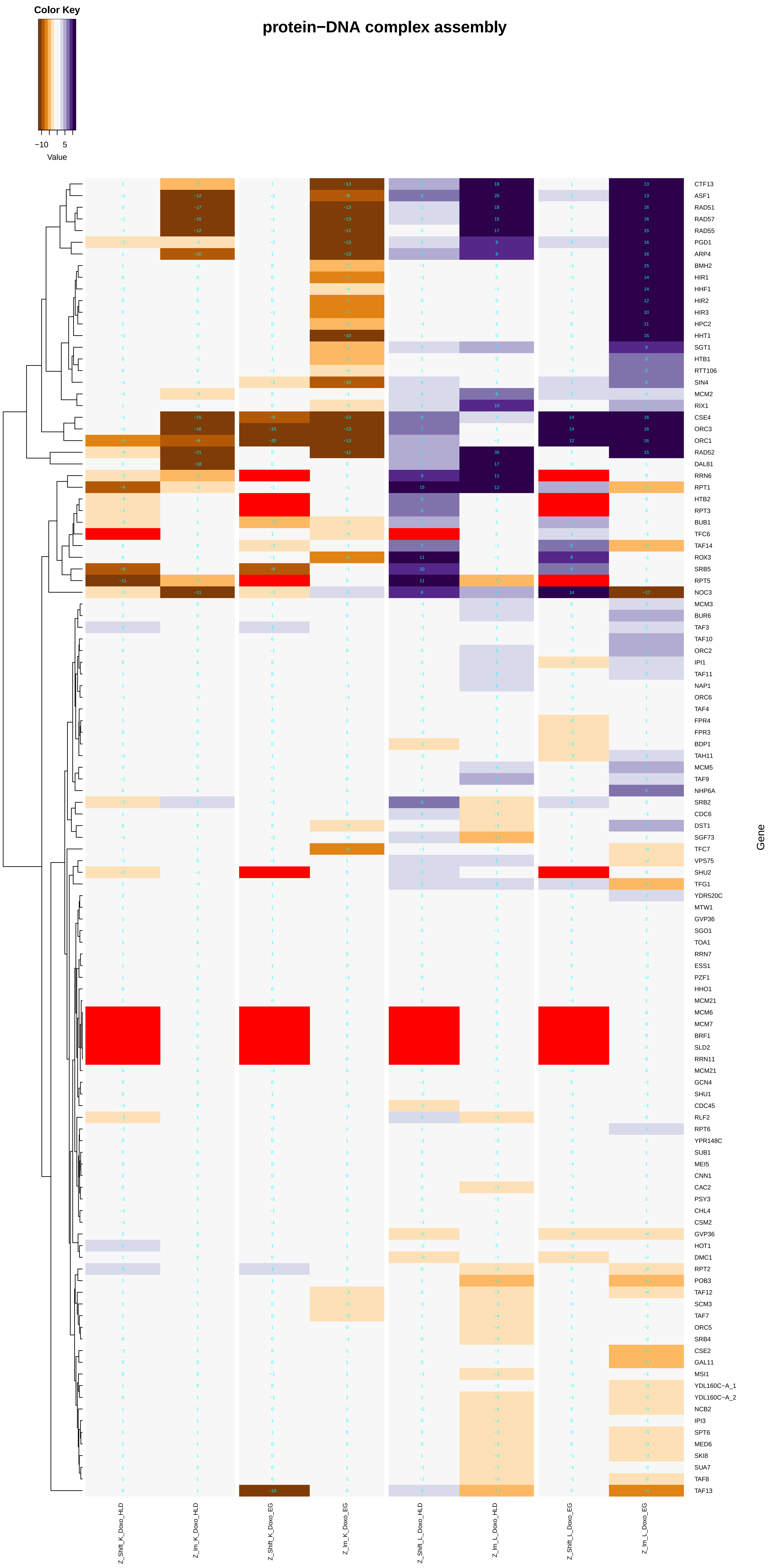

protein−DNA complex assembly
Color Key
−10
5
Value
Gene
CTF13
ASF1
RAD51
RAD57
RAD55
PGD1
ARP4
BMH2
HIR1
HHF1
HIR2
HIR3
HPC2
HHT1
SGT1
HTB1
RTT106
SIN4
MCM2
RIX1
CSE4
ORC3
ORC1
RAD52
DAL81
RRN6
RPT1
HTB2
RPT3
BUB1
TFC6
TAF14
ROX3
SRB5
RPT5
NOC3
MCM3
BUR6
TAF3
TAF10
ORC2
IPI1
TAF11
NAP1
ORC6
TAF4
FPR4
FPR3
BDP1
TAH11
MCM5
TAF9
NHP6A
SRB2
CDC6
DST1
SGF73
TFC7
VPS75
SHU2
TFG1
YDR520C
MTW1
GVP36
SGO1
TOA1
RRN7
ESS1
PZF1
HHO1
MCM21
MCM6
MCM7
BRF1
SLD2
RRN11
MCM21
GCN4
SHU1
CDC45
RLF2
RPT6
YPR148C
SUB1
MEI5
CNN1
CAC2
PSY3
CHL4
CSM2
GVP36
HOT1
DMC1
RPT2
POB3
TAF12
SCM3
TAF7
ORC5
SRB4
CSE2
GAL11
MSI1
YDL160C−A_1
YDL160C−A_2
NCB2
IPI3
SPT6
MED6
SKI8
SUA7
TAF8
TAF13
Z_Shift_K_Doxo_HLD
Z_lm_K_Doxo_HLD
Z_Shift_K_Doxo_EG
Z_lm_K_Doxo_EG
Z_Shift_L_Doxo_HLD
Z_lm_L_Doxo_HLD
Z_Shift_L_Doxo_EG
Z_lm_L_Doxo_EG

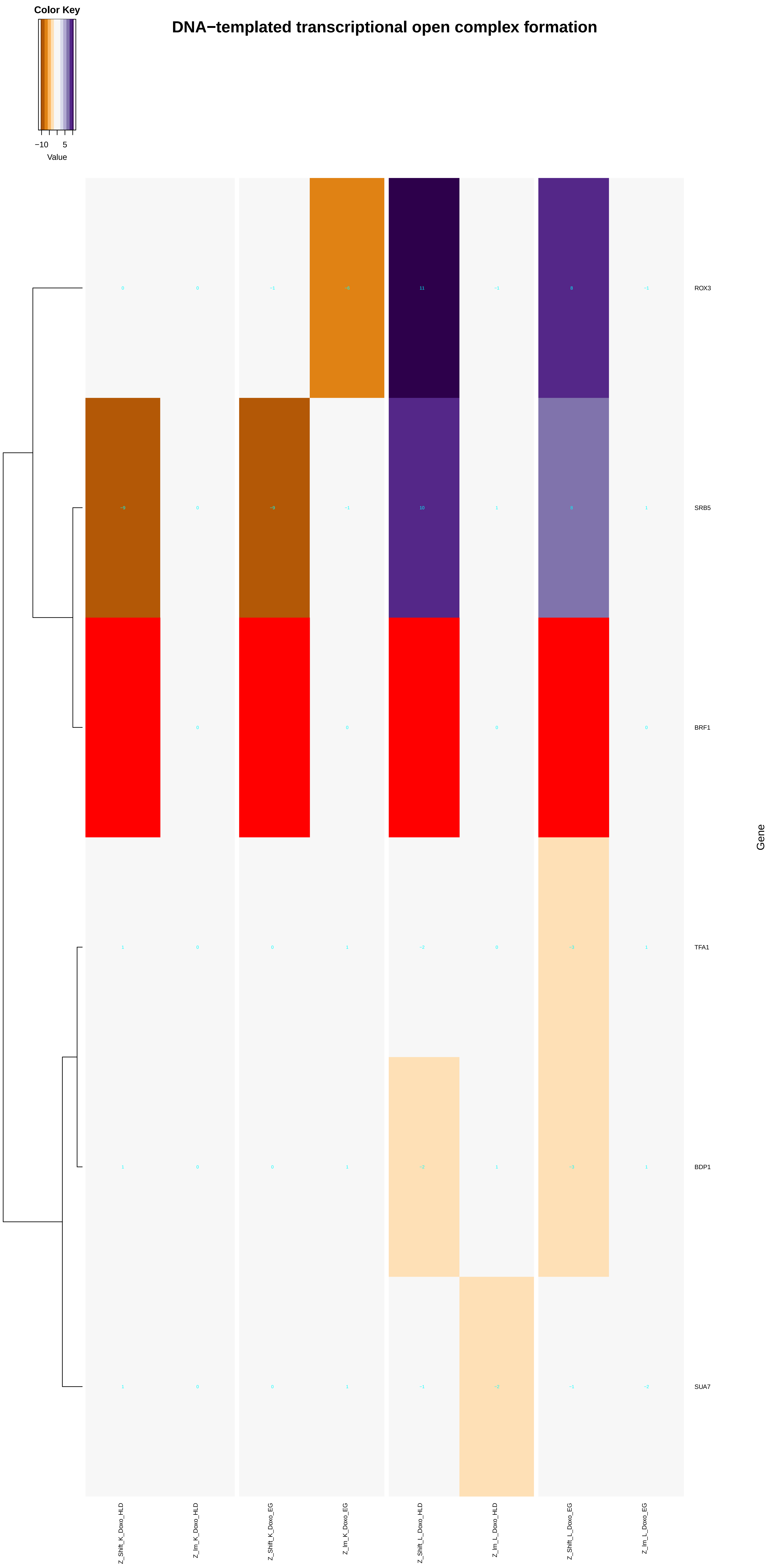
DNA−templated transcriptional open complex formation
Color Key
−10
5
Value
ROX3
SRB5
BRF1
TFA1
BDP1
SUA7
Gene
Z_Shift_K_Doxo_HLD
Z_Im_K_Doxo_HLD
Z_Shift_K_Doxo_EG
Z_Im_K_Doxo_EG
Z_Shift_L_Doxo_HLD
Z_Im_L_Doxo_HLD
Z_Shift_L_Doxo_EG
Z_Im_L_Doxo_EG

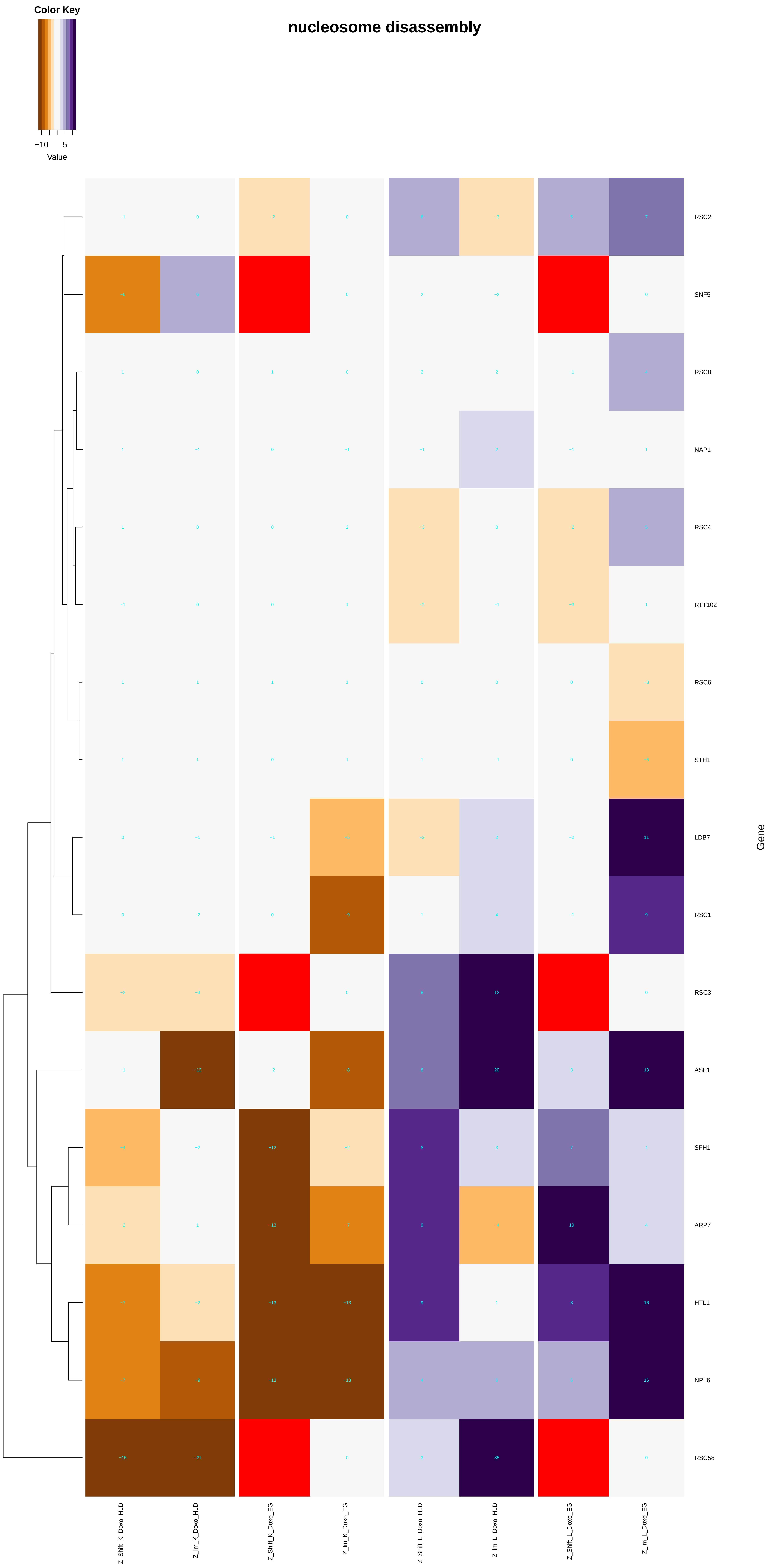
Color Key
−10
5
Value
nucleosome disassembly
RSC2
SNF5
RSC8
NAP1
RSC4
RTT102
RSC6
STH1
LDB7
RSC1
RSC3
ASF1
SFH1
ARP7
HTL1
NPL6
RSC58
Gene
Z_Shift_K_Doxo_HLD
Z_Im_K_Doxo_HLD
Z_Shift_K_Doxo_EG
Z_Im_K_Doxo_EG
Z_Shift_L_Doxo_HLD
Z_Im_L_Doxo_HLD
Z_Shift_L_Doxo_EG
Z_Im_L_Doxo_EG

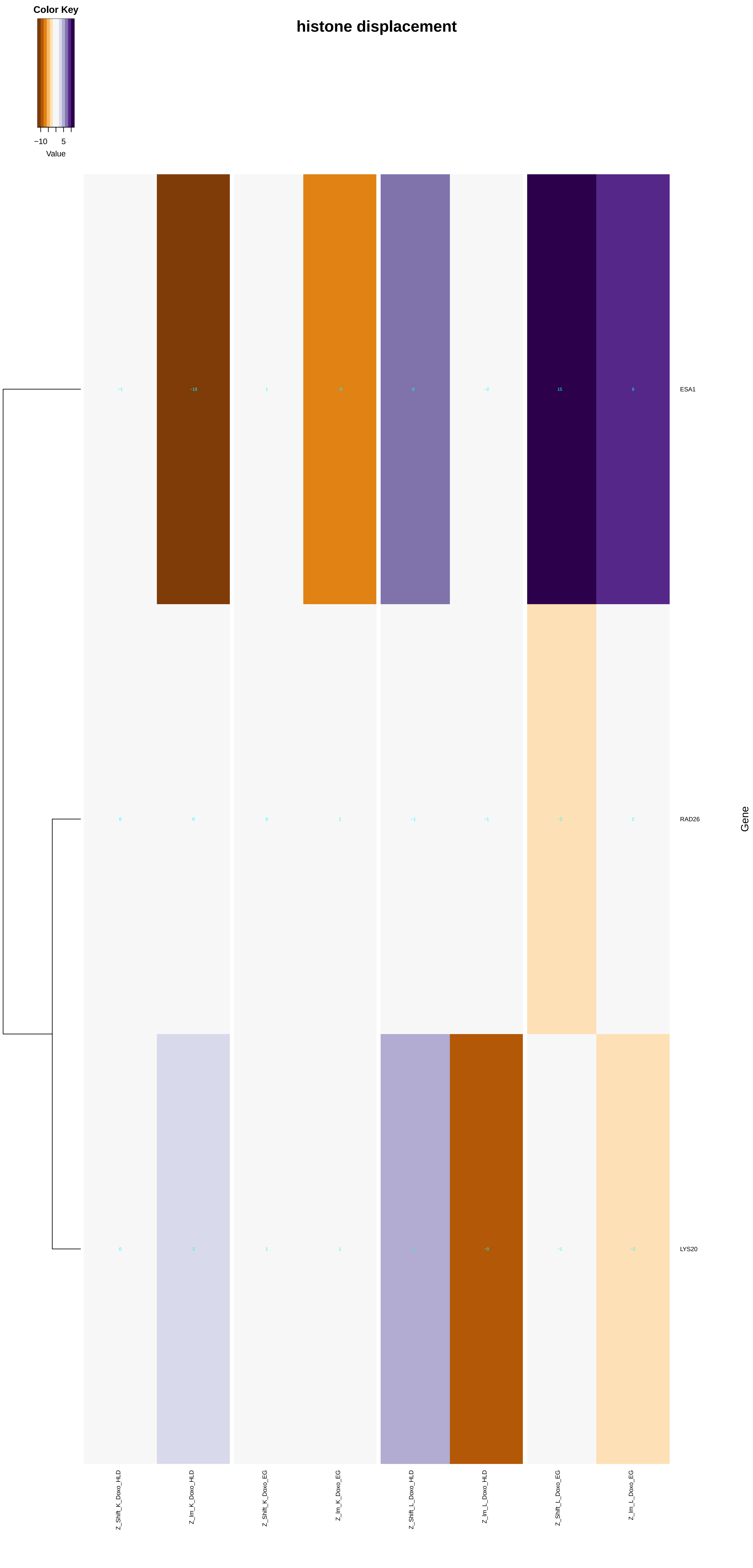
Color Key
−10
5
Value
histone displacement
−1
−18
1
−8
8
−2
15
9
ESA1
0
0
0
1
−1
−1
−2
2
RAD26
0
3
1
1
−9
−1
−2
LYS20
Gene
Z_Shift_K_Doxo_HLD
Z_lm_K_Doxo_HLD
Z_Shift_K_Doxo_EG
Z_lm_K_Doxo_EG
Z_Shift_L_Doxo_HLD
Z_lm_L_Doxo_HLD
Z_Shift_L_Doxo_EG
Z_lm_L_Doxo_EG

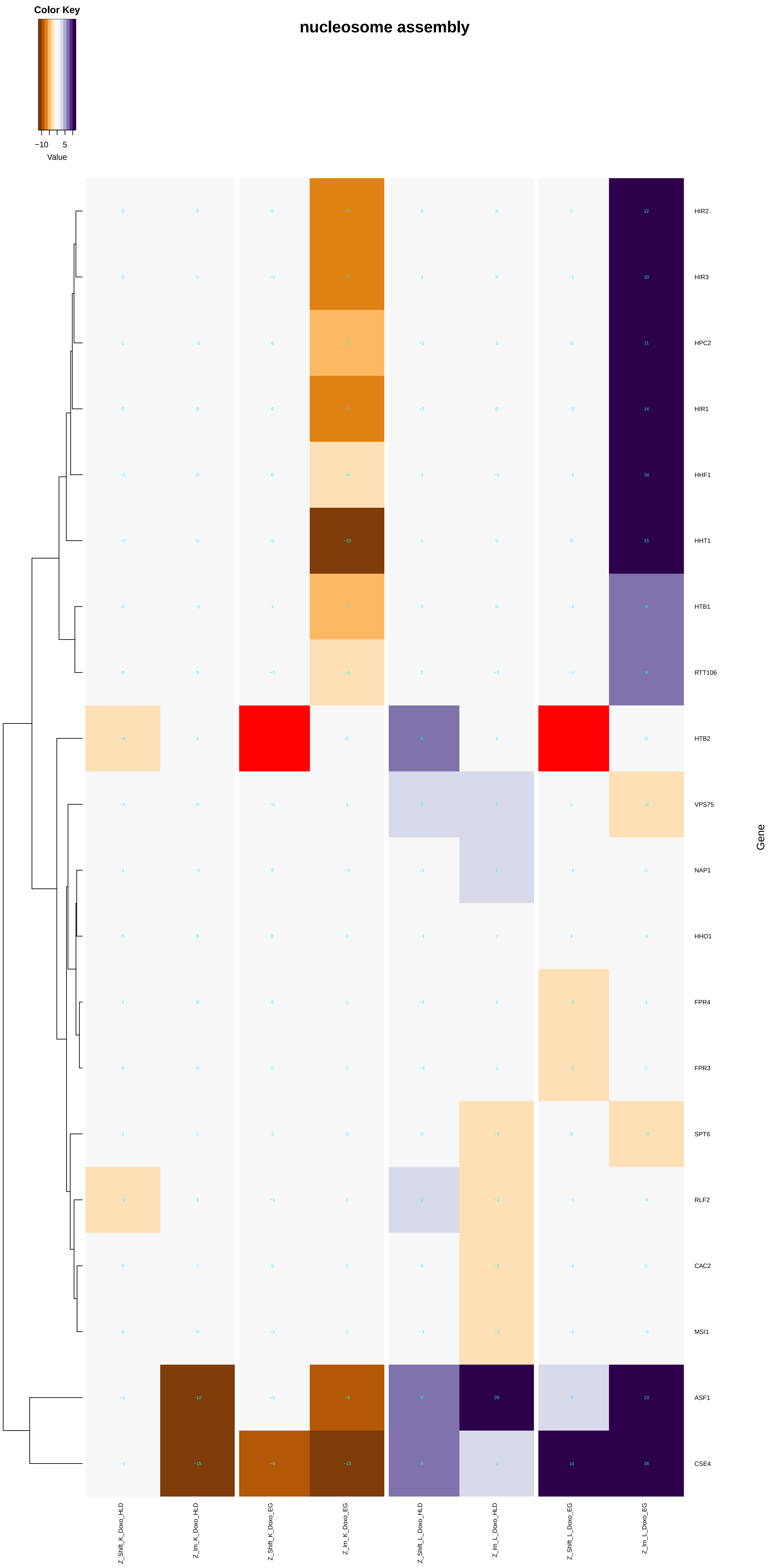
nucleosome assembly
Color Key
−10
5
Value
HIR2
HIR3
HPC2
HIR1
HHF1
HHT1
HTB1
RTT106
HTB2
VPS75
NAP1
HHO1
FPR4
FPR3
SPT6
RLF2
CAC2
MSI1
ASF1
CSE4
Gene
Z_Shift_K_Doxo_HLD
Z_lm_K_Doxo_HLD
Z_Shift_K_Doxo_EG
Z_lm_K_Doxo_EG
Z_Shift_L_Doxo_HLD
Z_lm_L_Doxo_HLD
Z_Shift_L_Doxo_EG
Z_lm_L_Doxo_EG

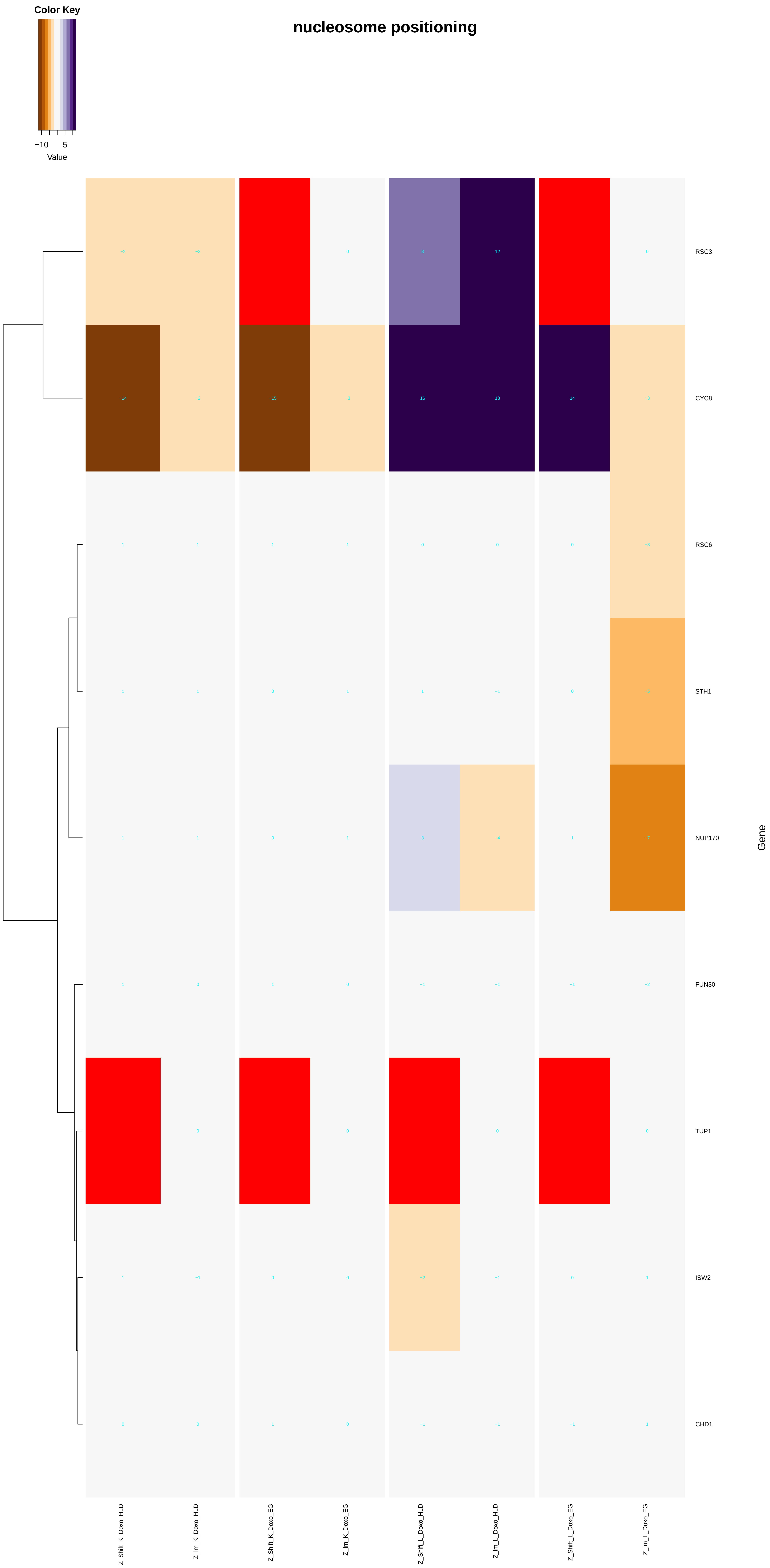
Color Key
−10
5
Value
nucleosome positioning
RSC3
CYC8
RSC6
STH1
NUP170
FUN30
TUP1
ISW2
CHD1
Gene
Z_Shift_K_Doxo_HLD
Z_lm_K_Doxo_HLD
Z_Shift_K_Doxo_EG
Z_lm_K_Doxo_EG
Z_Shift_L_Doxo_HLD
Z_lm_L_Doxo_HLD
Z_Shift_L_Doxo_EG
Z_lm_L_Doxo_EG

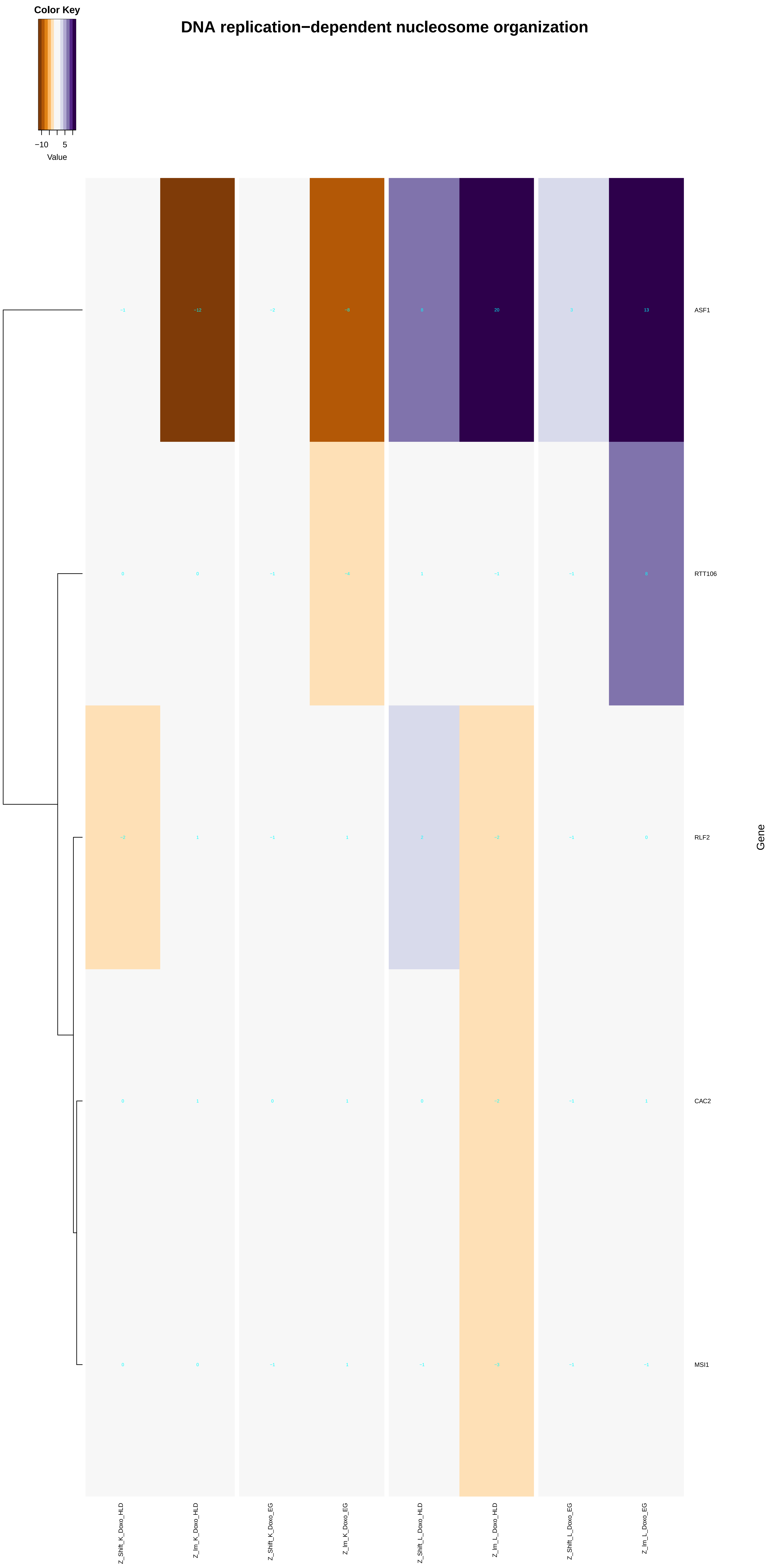
Color Key
−10
5
Value
DNA replication−dependent nucleosome organization
−1
−12
−2
−8
8
20
3
13
ASF1
0
0
−1
−4
1
−1
−1
8
RTT106
−2
1
−1
1
2
−2
−1
0
RLF2
0
1
0
1
0
−2
−1
1
CAC2
0
0
−1
1
−1
−3
−1
−1
MSI1
Gene
Z_Shift_K_Doxo_HLD
Z_lm_K_Doxo_HLD
Z_Shift_K_Doxo_EG
Z_lm_K_Doxo_EG
Z_Shift_L_Doxo_HLD
Z_lm_L_Doxo_HLD
Z_Shift_L_Doxo_EG
Z_lm_L_Doxo_EG

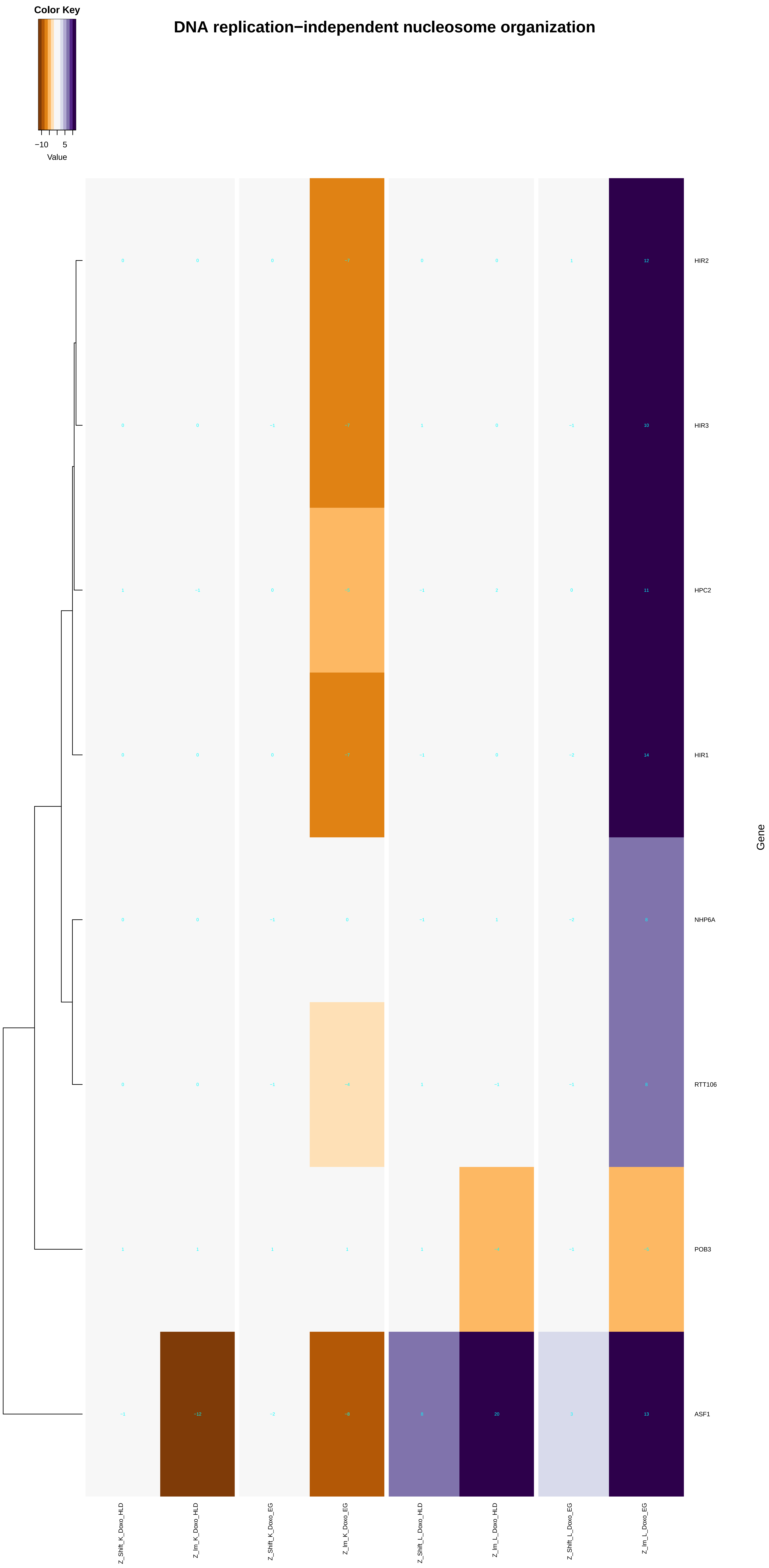
Color Key
−10
5
Value
DNA replication−independent nucleosome organization
HIR2
HIR3
HPC2
HIR1
NHP6A
RTT106
POB3
ASF1
Gene
Z_Shift_K_Doxo_HLD
Z_Im_K_Doxo_HLD
Z_Shift_K_Doxo_EG
Z_Im_K_Doxo_EG
Z_Shift_L_Doxo_HLD
Z_Im_L_Doxo_HLD
Z_Shift_L_Doxo_EG
Z_Im_L_Doxo_EG

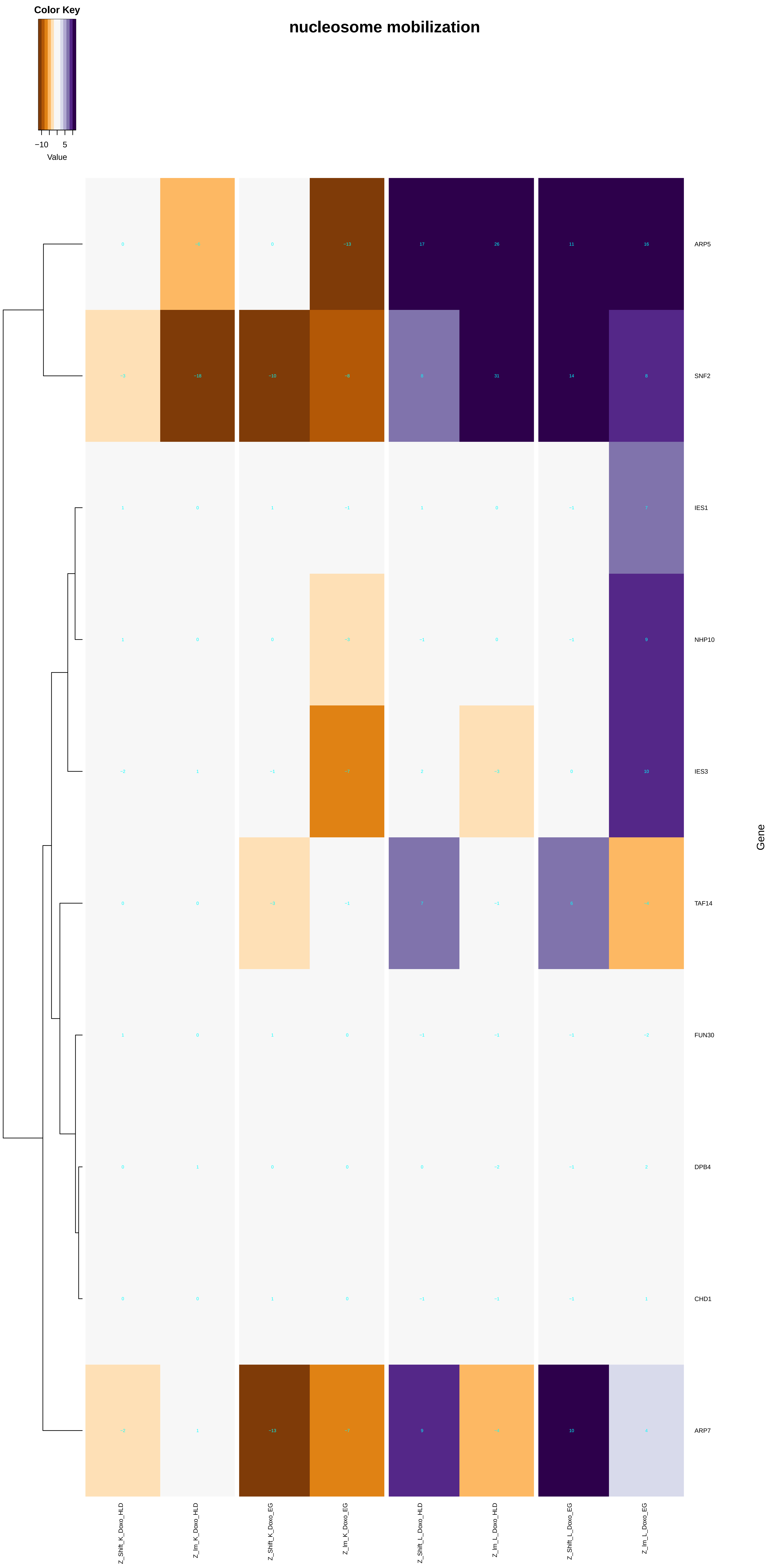
nucleosome mobilization
Color Key
−10
5
Value
ARP5
SNF2
IES1
NHP10
IES3
TAF14
FUN30
DPB4
CHD1
ARP7
Gene
Z_Shift_K_Doxo_HLD
Z_Im_K_Doxo_HLD
Z_Shift_K_Doxo_EG
Z_Im_K_Doxo_EG
Z_Shift_L_Doxo_HLD
Z_Im_L_Doxo_HLD
Z_Shift_L_Doxo_EG
Z_Im_L_Doxo_EG

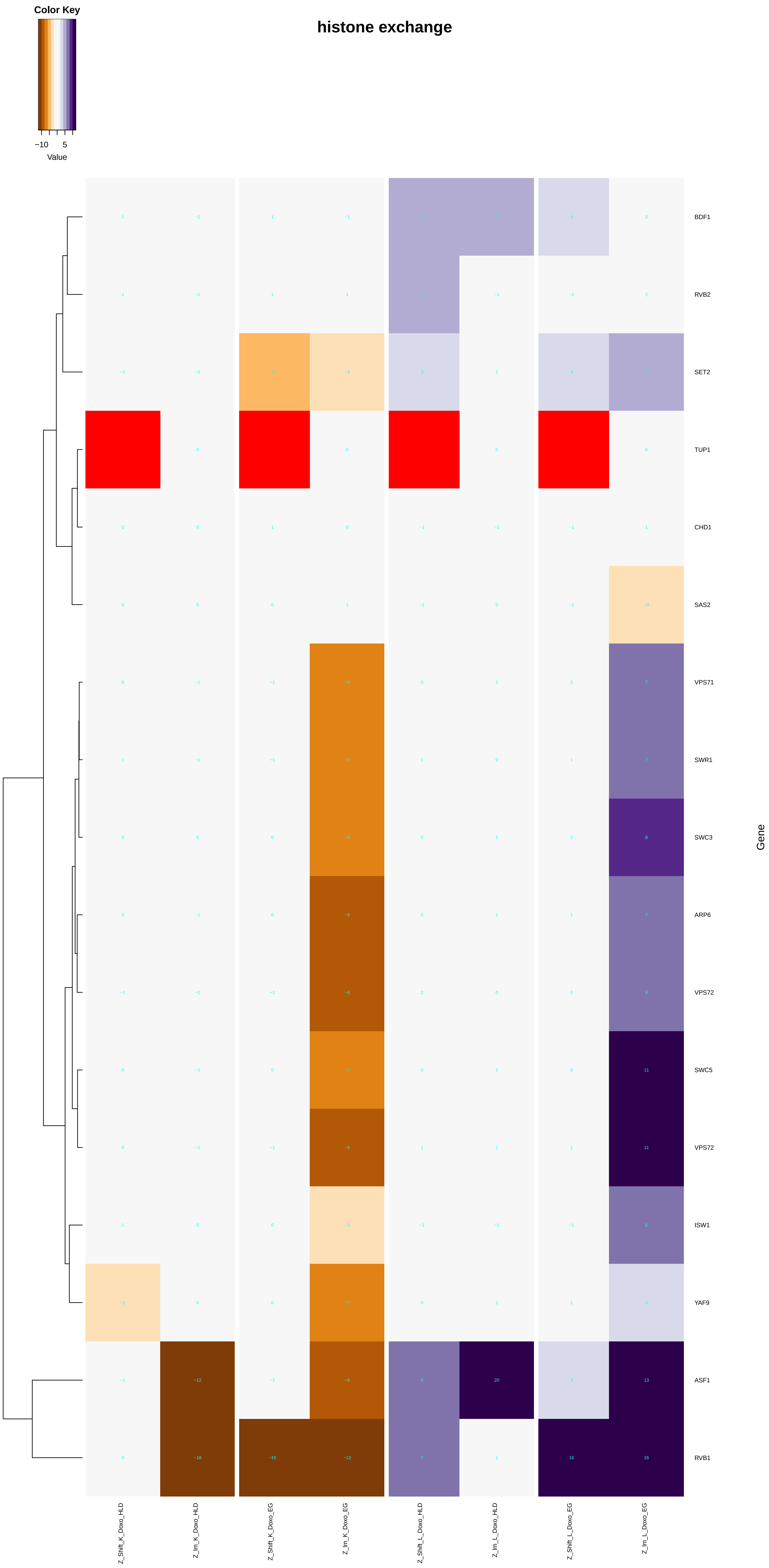
Color Key
−10
5
Value
histone exchange
BDF1
RVB2
SET2
TUP1
CHD1
SAS2
VPS71
SWR1
SWC3
ARP6
VPS72
SWC5
VPS72
ISW1
YAF9
ASF1
RVB1
Gene
Z_Shift_K_Doxo_HLD
Z_Im_K_Doxo_HLD
Z_Shift_K_Doxo_EG
Z_Im_K_Doxo_EG
Z_Shift_L_Doxo_HLD
Z_Im_L_Doxo_HLD
Z_Shift_L_Doxo_EG
Z_Im_L_Doxo_EG

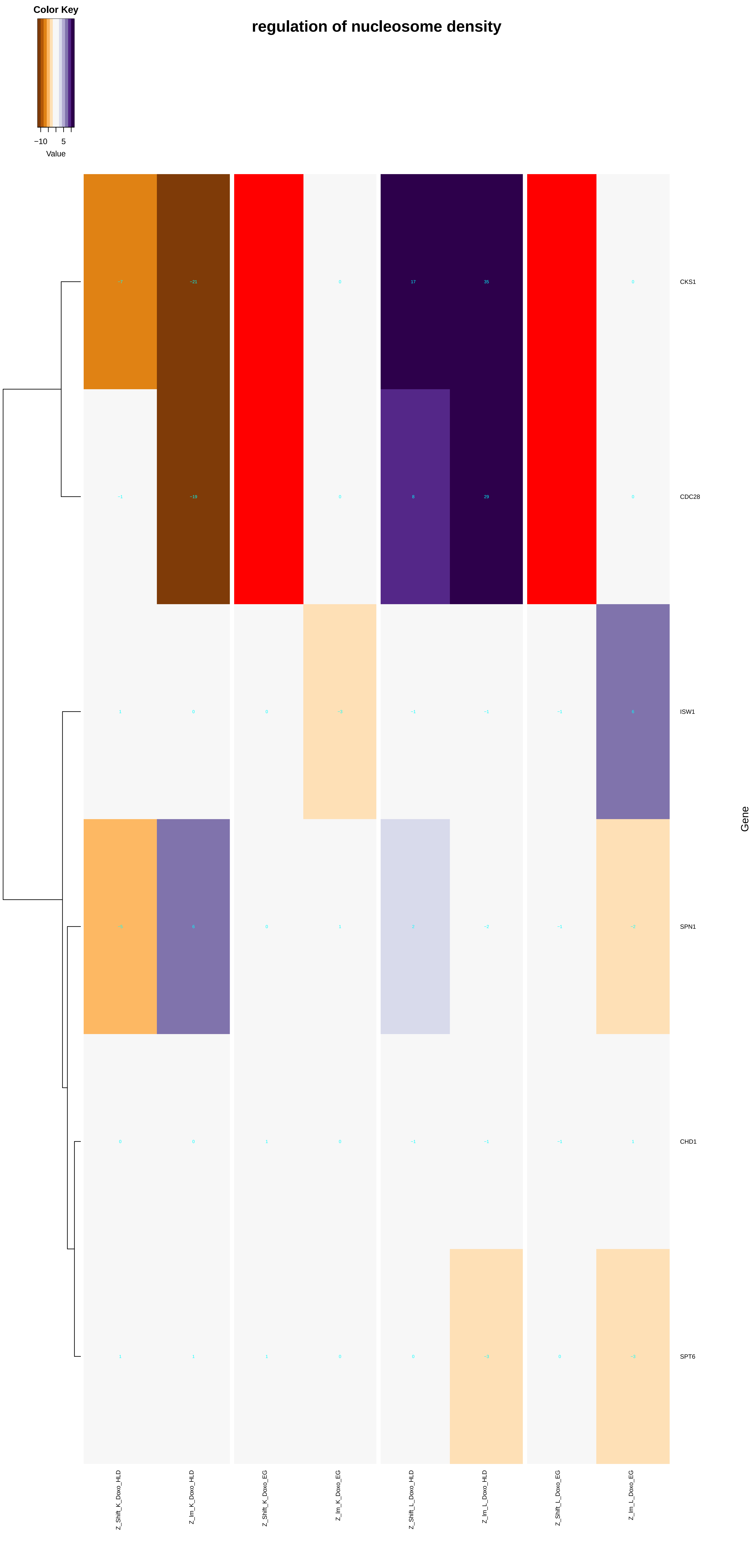
Color Key
−10
5
Value
regulation of nucleosome density
CKS1
CDC28
ISW1
SPN1
CHD1
SPT6
Gene
Z_Shift_K_Doxo_HLD
Z_Im_K_Doxo_HLD
Z_Shift_K_Doxo_EG
Z_Im_K_Doxo_EG
Z_Shift_L_Doxo_HLD
Z_Im_L_Doxo_HLD
Z_Shift_L_Doxo_EG
Z_Im_L_Doxo_EG

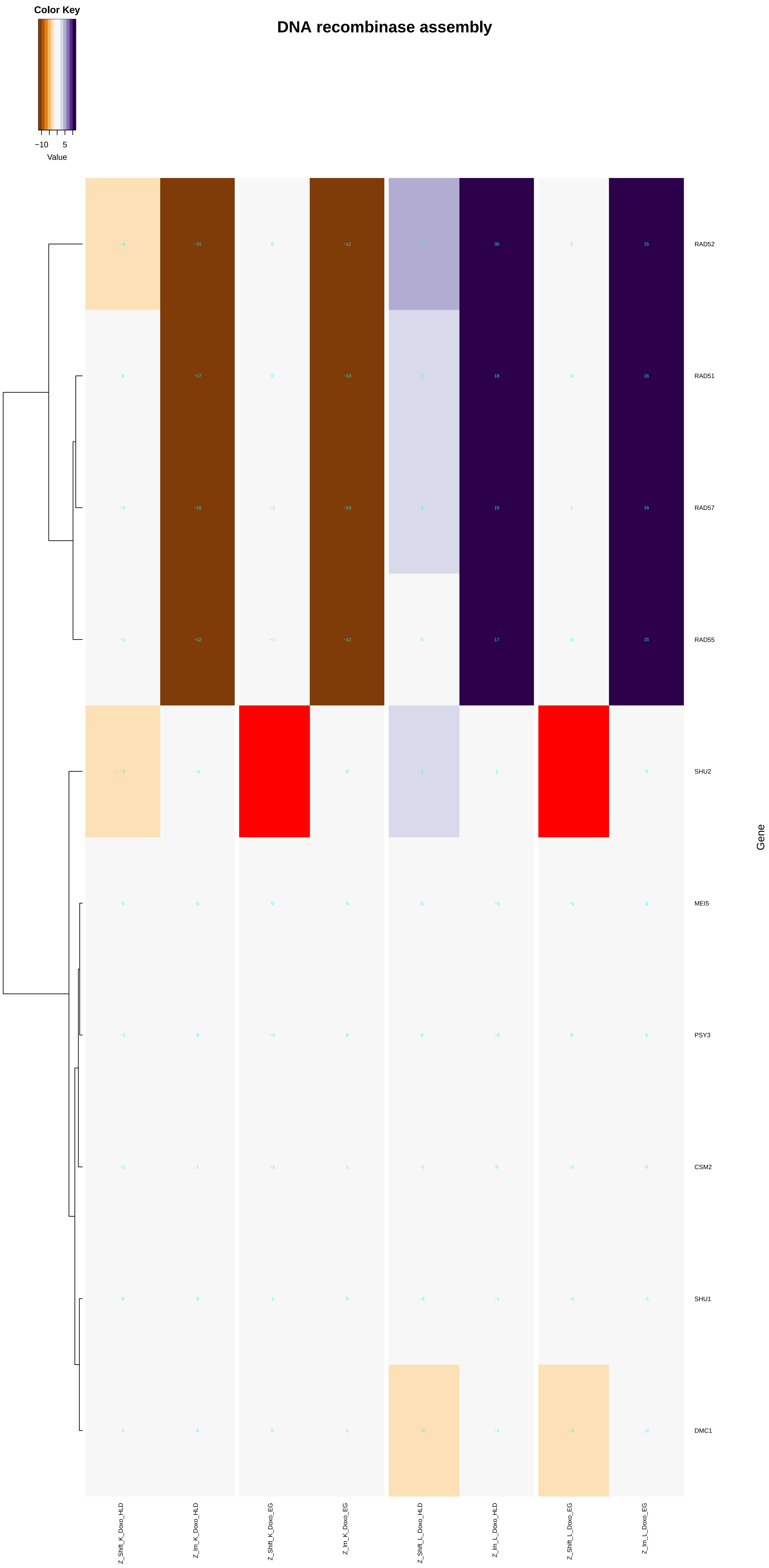
Color Key
−10
5
Value
DNA recombinase assembly
RAD52
RAD51
RAD57
RAD55
SHU2
MEI5
PSY3
CSM2
SHU1
DMC1
Gene
Z_Shift_K_Doxo_HLD
Z_Im_K_Doxo_HLD
Z_Shift_K_Doxo_EG
Z_Im_K_Doxo_EG
Z_Shift_L_Doxo_HLD
Z_Im_L_Doxo_HLD
Z_Shift_L_Doxo_EG
Z_Im_L_Doxo_EG

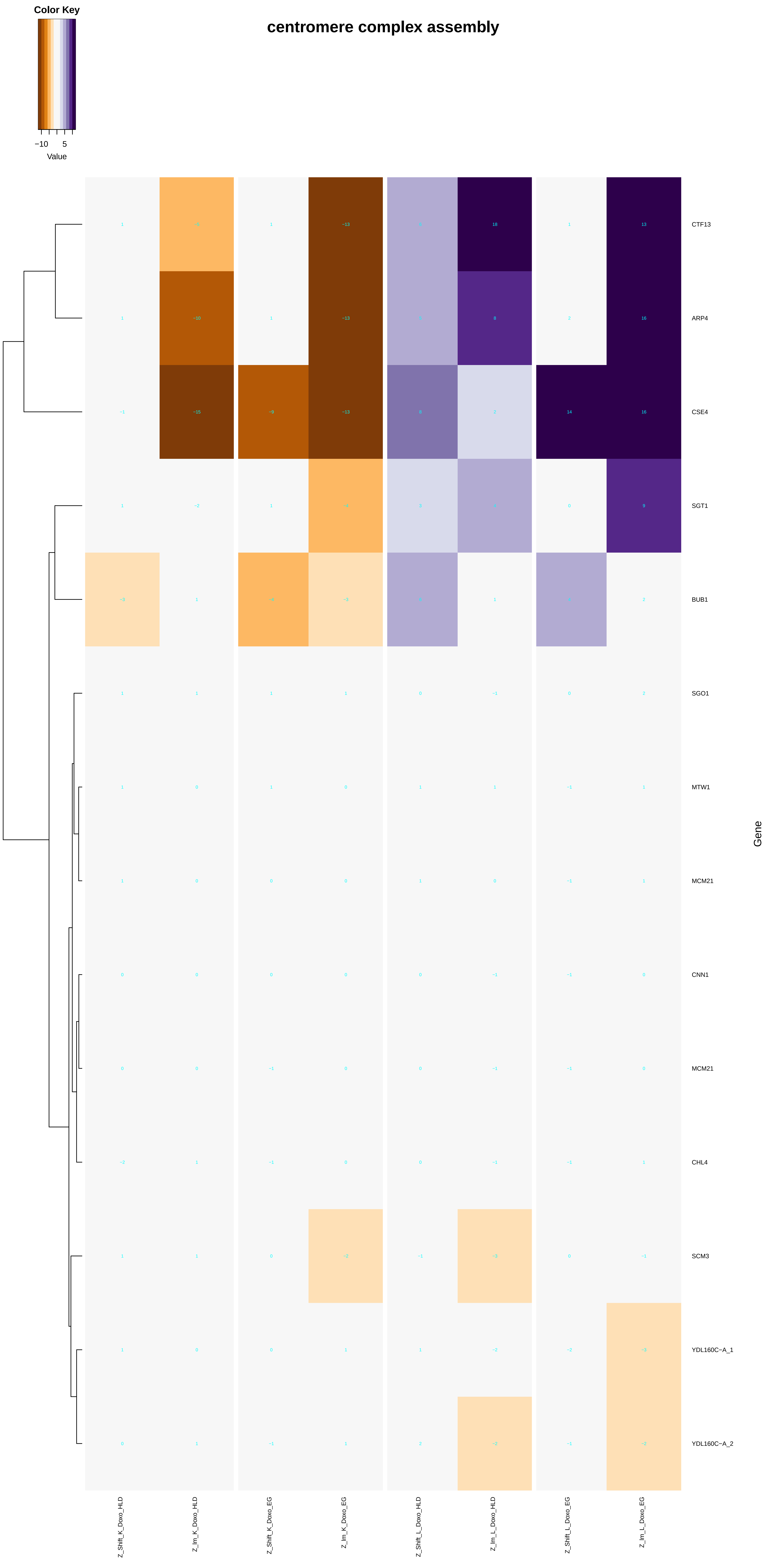
centromere complex assembly
Color Key
−10
5
Value
Gene
CTF13
ARP4
CSE4
SGT1
BUB1
SGO1
MTW1
MCM21
CNN1
MCM21
CHL4
SCM3
YDL160C−A_1
YDL160C−A_2
Z_Shift_K_Doxo_HLD
Z_Im_K_Doxo_HLD
Z_Shift_K_Doxo_EG
Z_Im_K_Doxo_EG
Z_Shift_L_Doxo_HLD
Z_Im_L_Doxo_HLD
Z_Shift_L_Doxo_EG
Z_Im_L_Doxo_EG

pre−replicative complex assembly

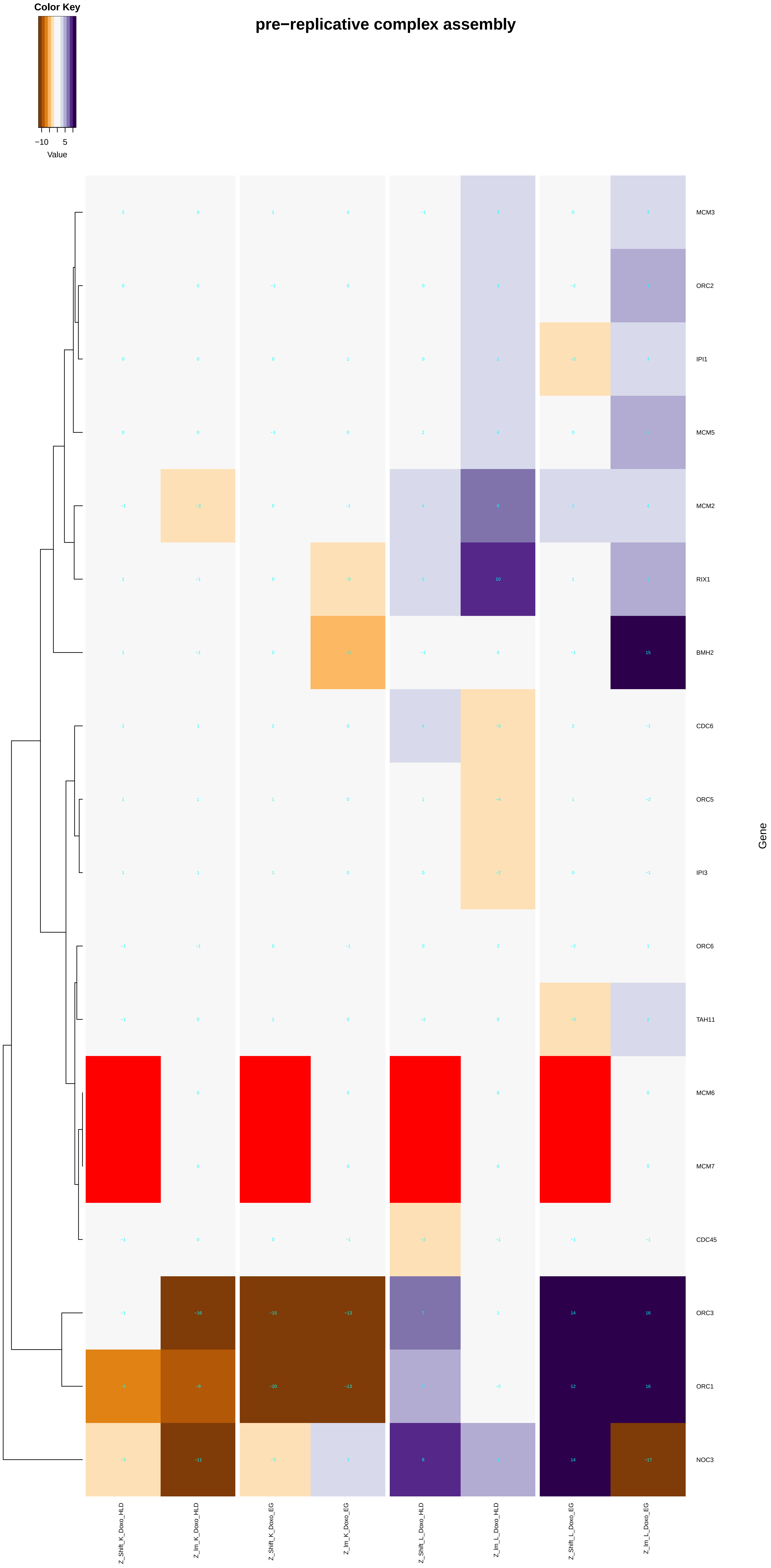

DNA−templated transcriptional preinitiation complex assembly

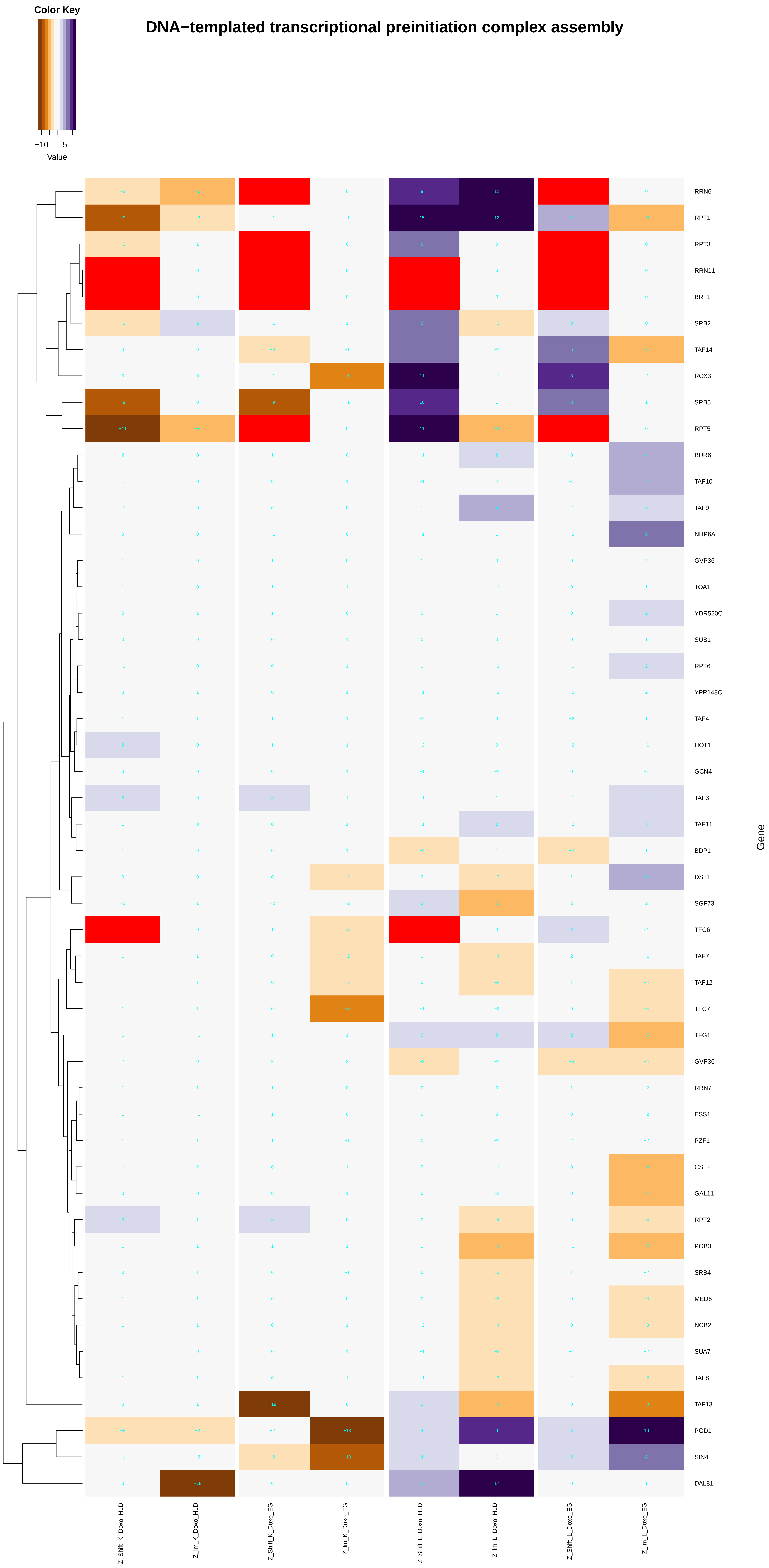

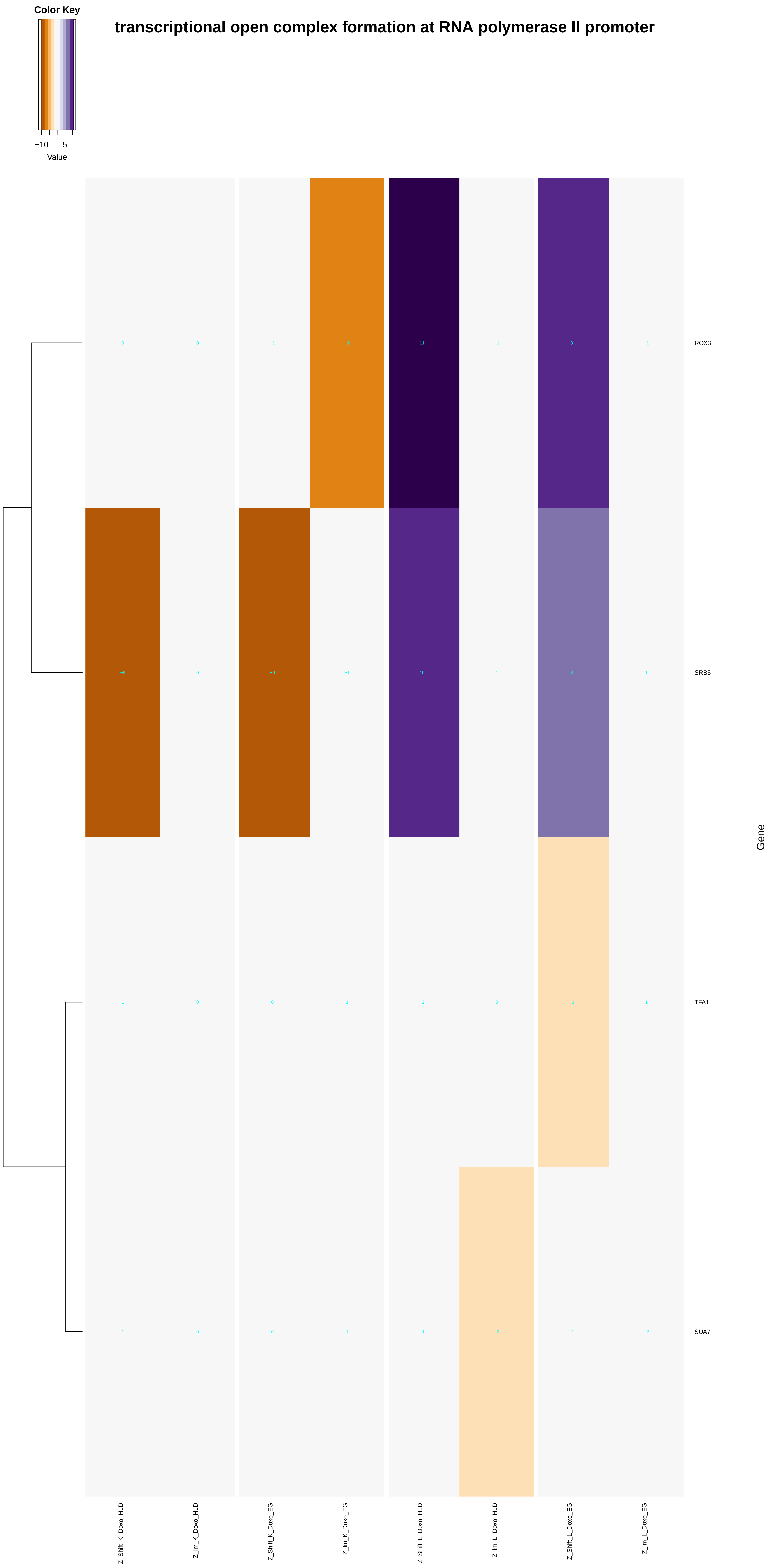

Color Key
−10
5
Value
transcriptional open complex formation at RNA polymerase II promoter
0
0
−1
−6
11
−1
8
−1
ROX3
−9
0
−9
−1
10
1
8
1
SRB5
1
0
0
1
−2
0
−3
1
TFA1
1
0
0
1
−1
−2
−1
−2
SUA7
Gene
Z_Shift_K_Doxo_HLD
Z_Im_K_Doxo_HLD
Z_Shift_K_Doxo_EG
Z_Im_K_Doxo_EG
Z_Shift_L_Doxo_HLD
Z_Im_L_Doxo_HLD
Z_Shift_L_Doxo_EG
Z_Im_L_Doxo_EG

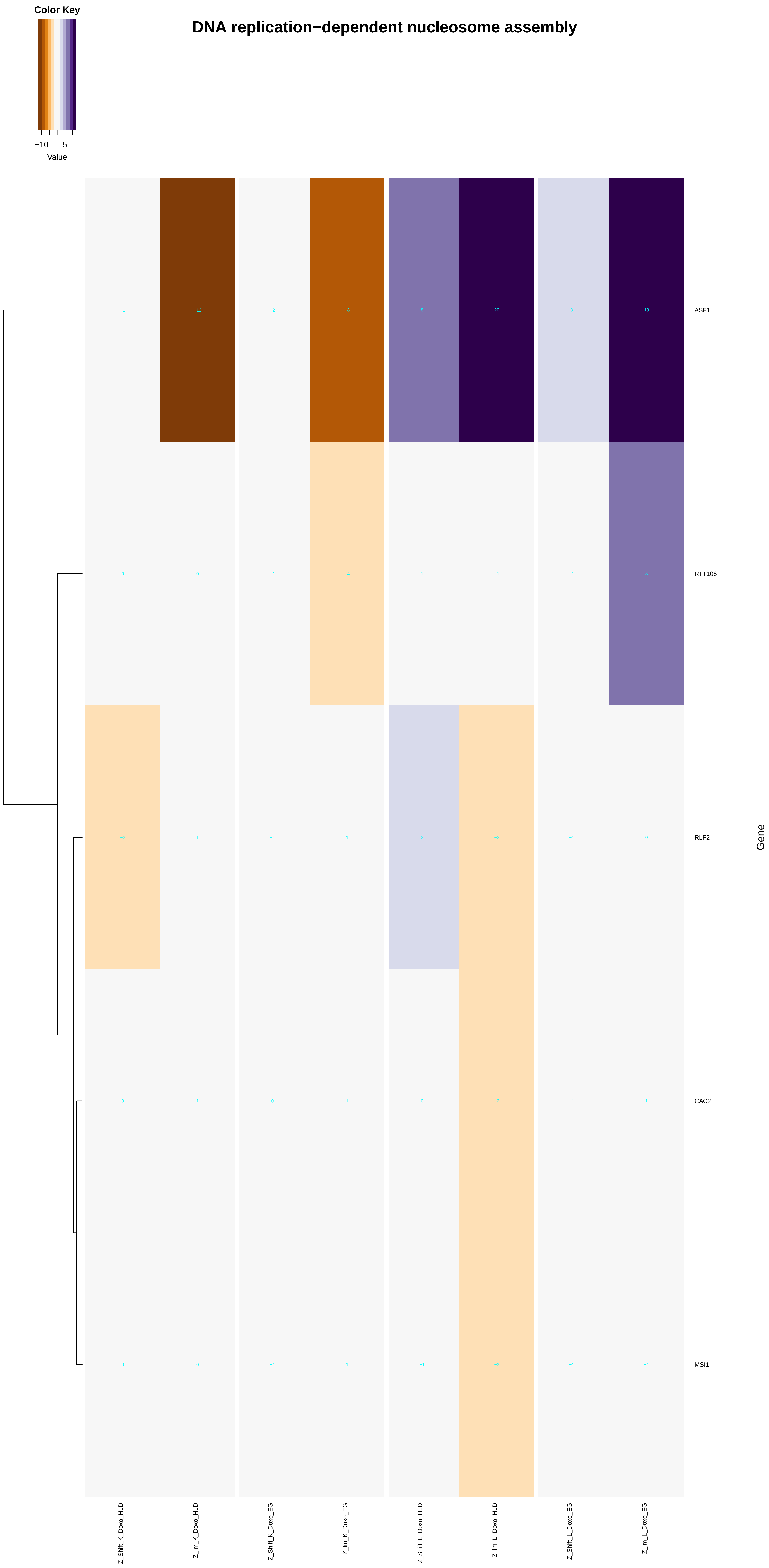
Color Key
−10
5
Value
DNA replication−dependent nucleosome assembly
−1
−12
−2
−8
8
20
3
13
ASF1
0
0
−1
−4
1
−1
−1
8
RTT106
−2
1
−1
1
2
−2
−1
0
RLF2
0
1
0
1
0
−2
−1
1
CAC2
0
0
−1
1
−1
−3
−1
−1
MSI1
Gene
Z_Shift_K_Doxo_HLD
Z_lm_K_Doxo_HLD
Z_Shift_K_Doxo_EG
Z_lm_K_Doxo_EG
Z_Shift_L_Doxo_HLD
Z_lm_L_Doxo_HLD
Z_Shift_L_Doxo_EG
Z_lm_L_Doxo_EG

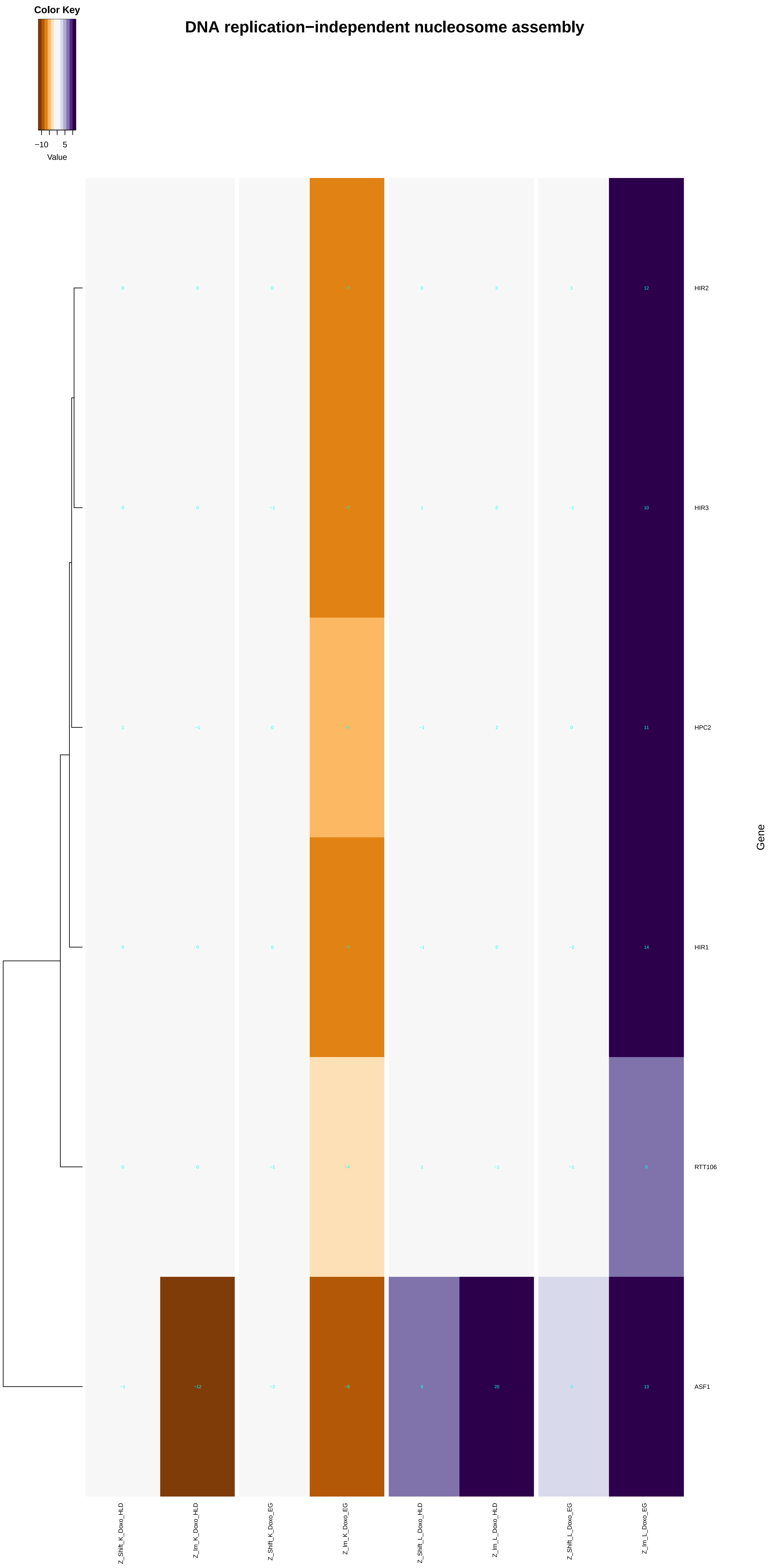
Color Key
−10
5
Value
DNA replication−independent nucleosome assembly
HIR2
HIR3
HPC2
HIR1
RTT106
ASF1
Gene
Z_Shift_K_Doxo_HLD
Z_Im_K_Doxo_HLD
Z_Shift_K_Doxo_EG
Z_Im_K_Doxo_EG
Z_Shift_L_Doxo_HLD
Z_Im_L_Doxo_HLD
Z_Shift_L_Doxo_EG
Z_Im_L_Doxo_EG

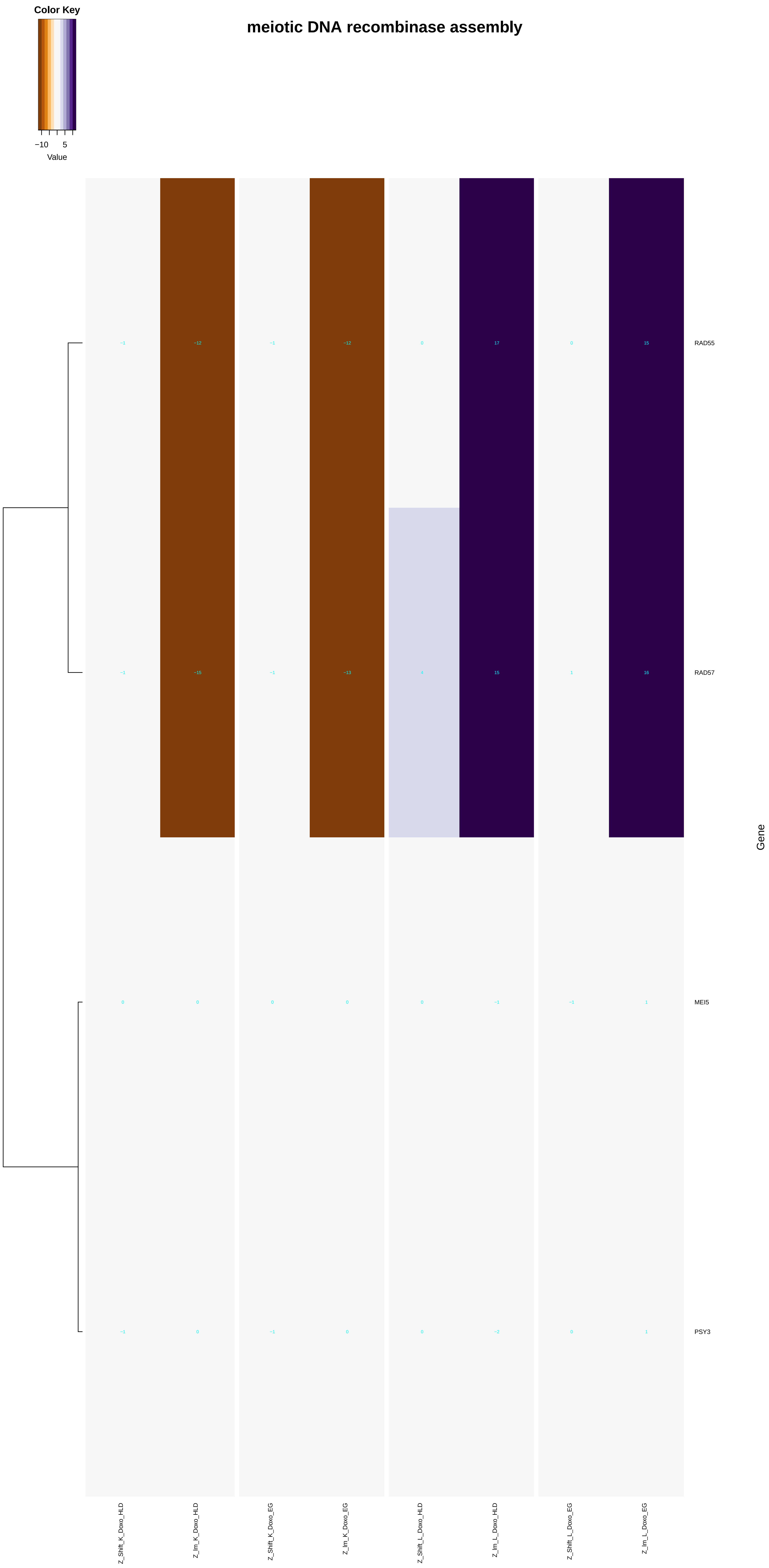
Color Key
−10
5
Value
meiotic DNA recombinase assembly
RAD55
RAD57
MEI5
PSY3
Gene
Z_Shift_K_Doxo_HLD
Z_lm_K_Doxo_HLD
Z_Shift_K_Doxo_EG
Z_lm_K_Doxo_EG
Z_Shift_L_Doxo_HLD
Z_lm_L_Doxo_HLD
Z_Shift_L_Doxo_EG
Z_lm_L_Doxo_EG

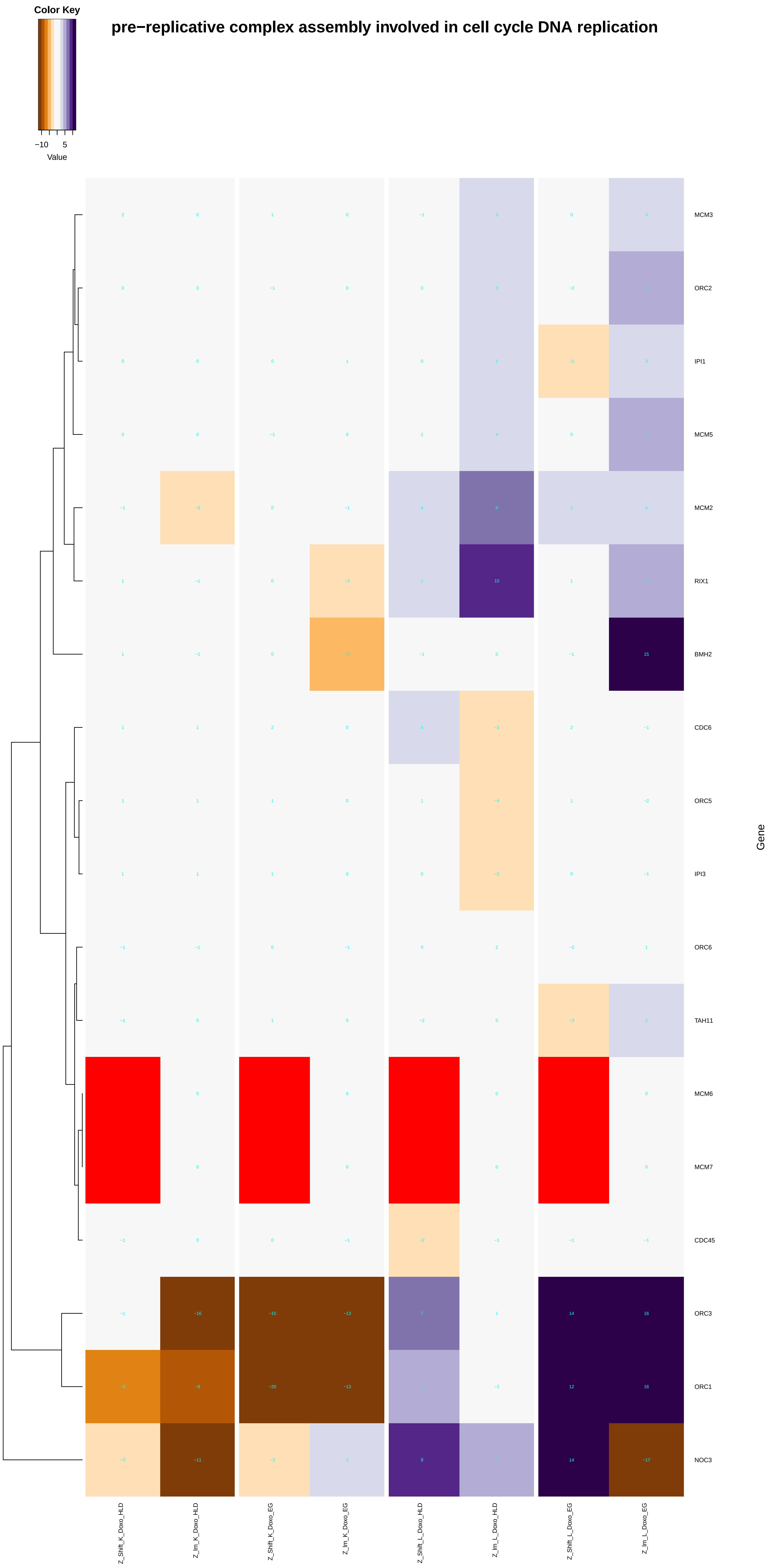
Color Key
−10
5
Value
pre−replicative complex assembly involved in cell cycle DNA replication
MCM3
ORC2
IPI1
MCM5
MCM2
RIX1
BMH2
CDC6
ORC5
IPI3
ORC6
TAH11
MCM6
MCM7
CDC45
ORC3
ORC1
NOC3
Gene
Z_Shift_K_Doxo_HLD
Z_Im_K_Doxo_HLD
Z_Shift_K_Doxo_EG
Z_Im_K_Doxo_EG
Z_Shift_L_Doxo_HLD
Z_Im_L_Doxo_HLD
Z_Shift_L_Doxo_EG
Z_Im_L_Doxo_EG

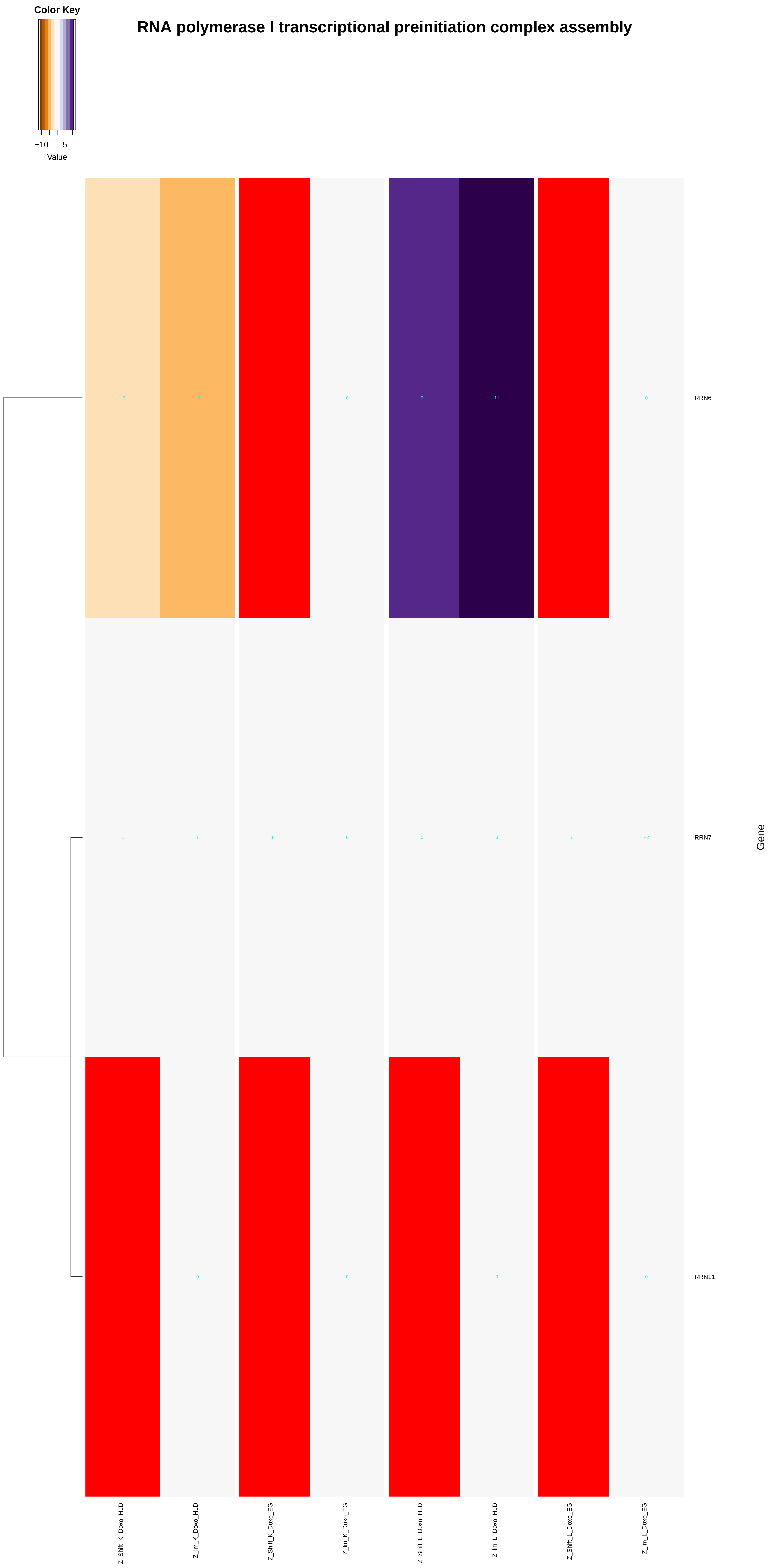

RNA polymerase I transcriptional preinitiation complex assembly
Color Key
−10
5
Value
−3
−6
0
9
11
0
RRN6
1
1
1
0
0
0
1
−2
RRN7
0
0
0
0
RRN11
Gene
Z_Shift_K_Doxo_HLD
Z_lm_K_Doxo_HLD
Z_Shift_K_Doxo_EG
Z_lm_K_Doxo_EG
Z_Shift_L_Doxo_HLD
Z_lm_L_Doxo_HLD
Z_Shift_L_Doxo_EG
Z_lm_L_Doxo_EG

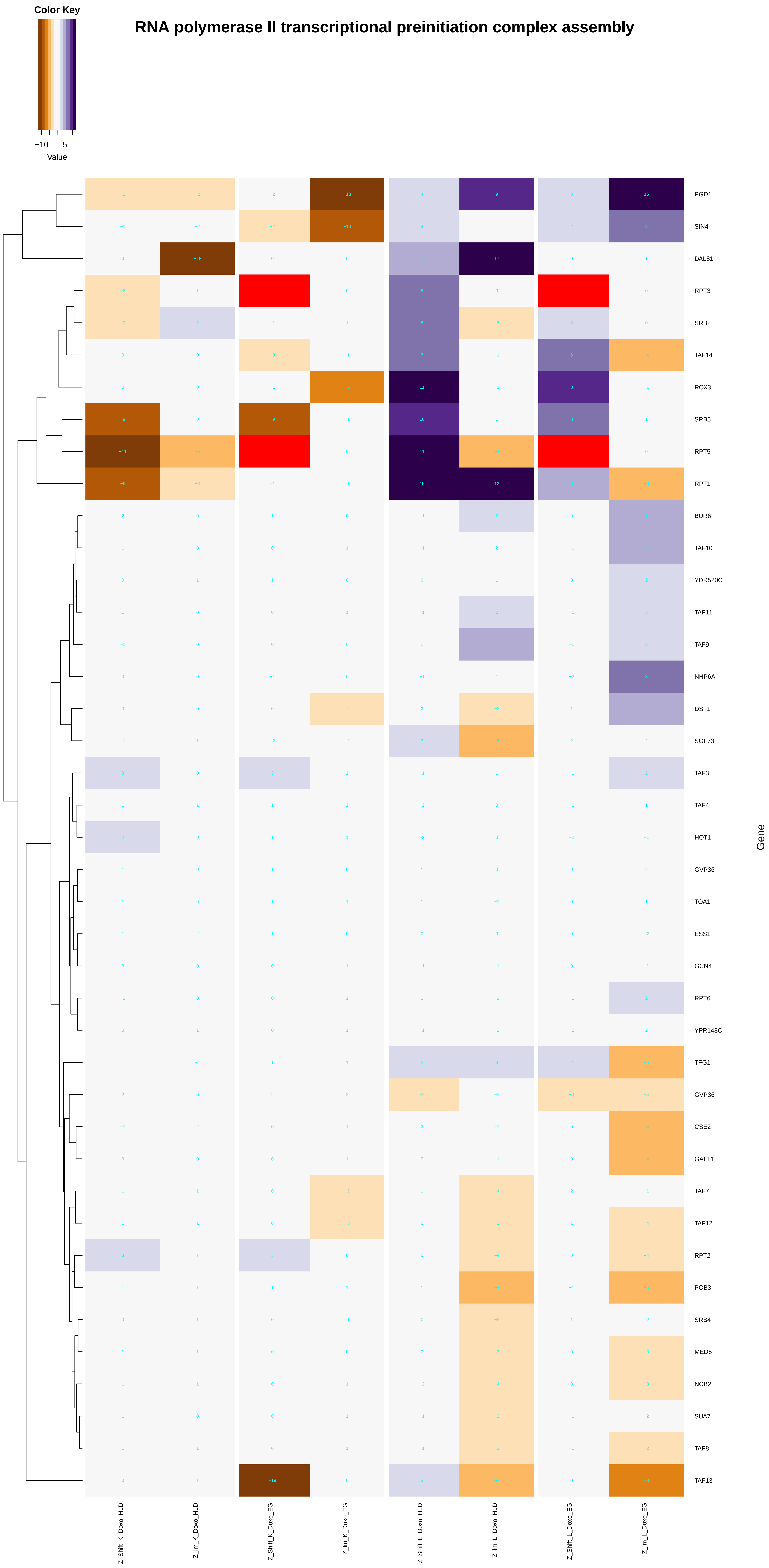

Color Key
−10 5
Value
RNA polymerase II transcriptional preinitiation complex assembly
PGD1
SIN4
DAL81
RPT3
SRB2
TAF14
ROX3
SRB5
RPT5
RPT1
BUR6
TAF10
YDR520C
TAF11
TAF9
NHP6A
DST1
SGF73
TAF3
TAF4
HOT1
GVP36
TOA1
ESS1
GCN4
RPT6
YPR148C
TFG1
GVP36
CSE2
GAL11
TAF7
TAF12
RPT2
POB3
SRB4
MED6
NCB2
SUA7
TAF8
TAF13
Gene
Z_Shift_K_Doxo_HLD
Z_Im_K_Doxo_HLD
Z_Shift_K_Doxo_EG
Z_Im_K_Doxo_EG
Z_Shift_L_Doxo_HLD
Z_Im_L_Doxo_HLD
Z_Shift_L_Doxo_EG
Z_Im_L_Doxo_EG

RNA polymerase III transcriptional preinitiation complex assembly

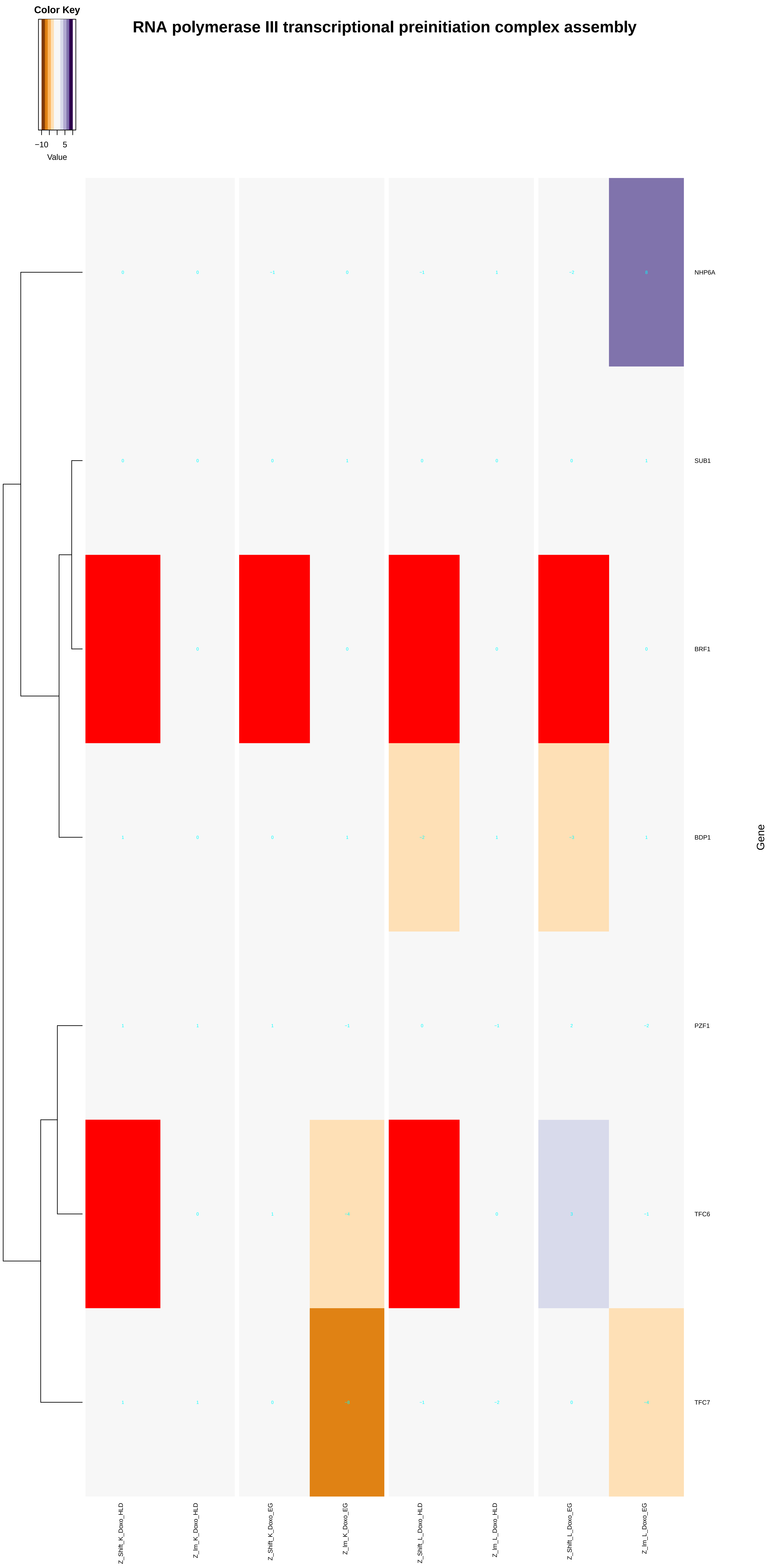

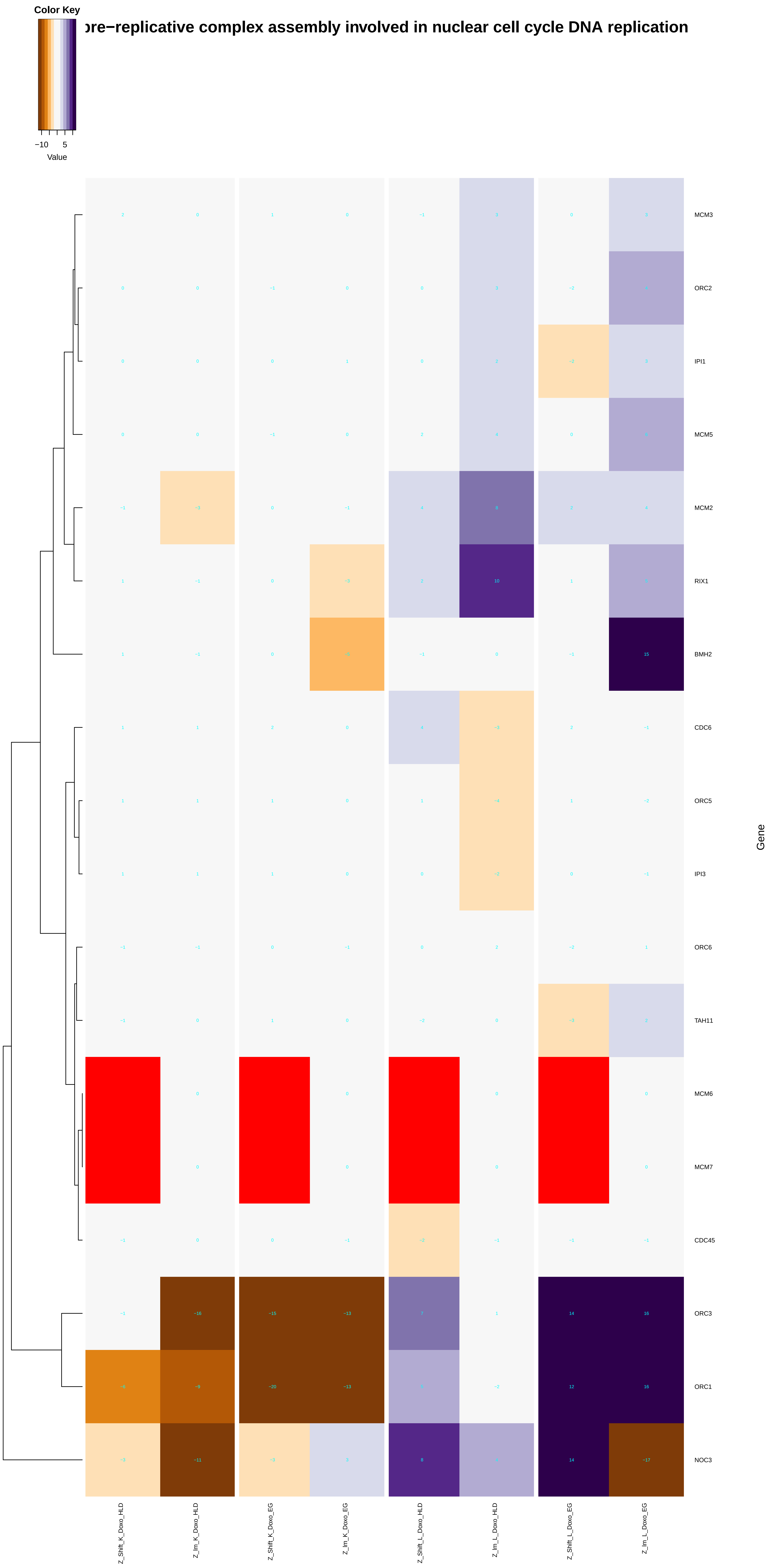
Color Key
−10
5
Value
re−replicative complex assembly involved in nuclear cell cycle DNA replication
MCM3
ORC2
IPI1
MCM5
MCM2
RIX1
BMH2
CDC6
ORC5
IPI3
ORC6
TAH11
MCM6
MCM7
CDC45
ORC3
ORC1
NOC3
Gene
Z_Shift_K_Doxo_HLD
Z_Im_K_Doxo_HLD
Z_Shift_K_Doxo_EG
Z_Im_K_Doxo_EG
Z_Shift_L_Doxo_HLD
Z_Im_L_Doxo_HLD
Z_Shift_L_Doxo_EG
Z_Im_L_Doxo_EG

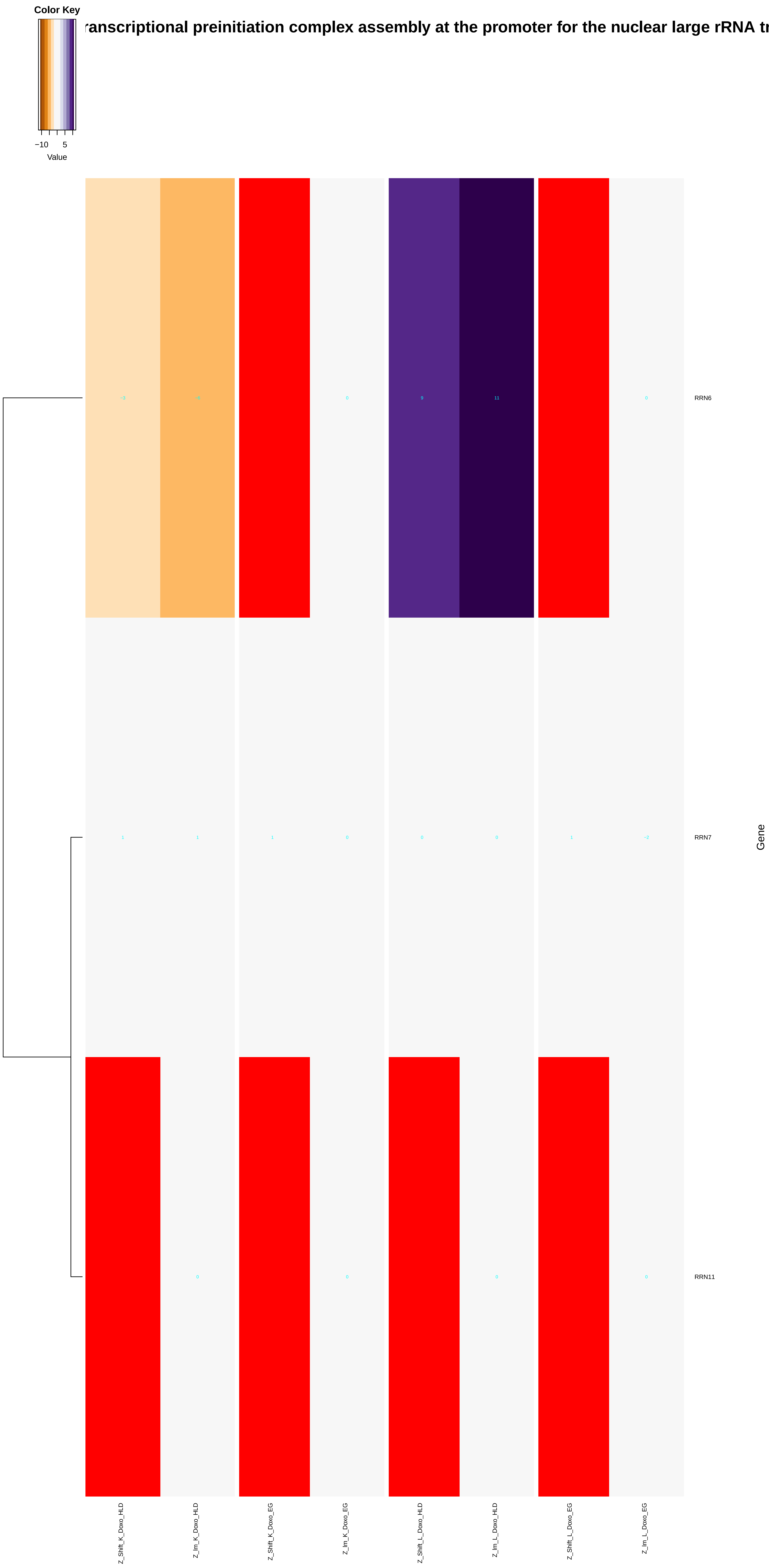

Color Key
−10
5
Value
ranscriptional preinitiation complex assembly at the promoter for the nuclear large rRNA tr
RRN6
RRN7
RRN11
Gene
Z_Shift_K_Doxo_HLD
Z_Im_K_Doxo_HLD
Z_Shift_K_Doxo_EG
Z_Im_K_Doxo_EG
Z_Shift_L_Doxo_HLD
Z_Im_L_Doxo_HLD
Z_Shift_L_Doxo_EG
Z_Im_L_Doxo_EG

NA polymerase III type 1 promoter transcriptional preinitiation complex assembly

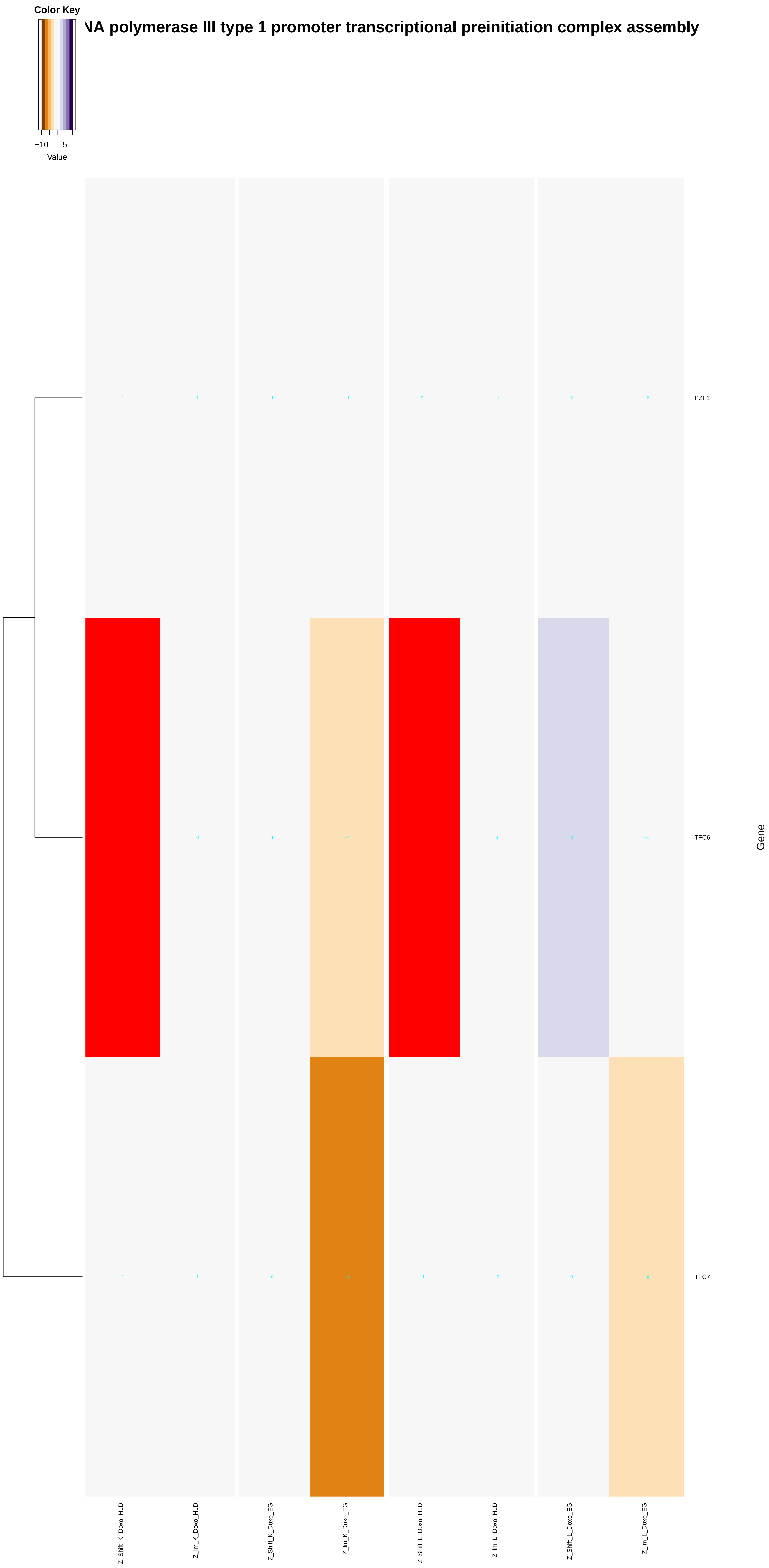

Supplement: Supplementary file 9 — Additional file 9. GO term-specific heatmaps for REMc/GTF-enriched clusters. GO term-specific heatmaps for significant GO process terms were generated as described in methods and Figs. 3 and 4. Any related child terms are presented in subsequent pages of the parent file name. GO terms with more than 100 children, with 2 or fewer genes annotated to the term, or a file size over 300KB are not shown. All heatmaps are generated with the same layout (see Figs. 3 and 4). [file 40170_2019_201_MOESM9_ESM.bz2 › Additional_File9_GOTermHeatmaps/Additional_File9_GOTermHeatmap/protein-DNA_complex_subunit_organization.pdf]
